# Toll-Like Receptor 7 Agonist RG7854 Mediates Therapeutic Efficacy and Seroconversion in Woodchucks With Chronic Hepatitis B

**DOI:** 10.3389/fimmu.2022.884113

**Published:** 2022-05-23

**Authors:** Steffen Wildum, Kyle E. Korolowicz, Manasa Suresh, Guido Steiner, Lue Dai, Bin Li, Changsuek Yon, Maria Cristina De Vera Mudry, Franziska Regenass-Lechner, Xu Huang, Xupeng Hong, Marta G. Murreddu, Bhaskar V. Kallakury, John A. T. Young, Stephan Menne

**Affiliations:** ^1^ Roche Pharma, Research and Early Development, Roche Innovation Center Basel, Basel, Switzerland; ^2^ Department of Microbiology and Immunology, Georgetown University Medical Center, Washington, DC, United States; ^3^ Roche Pharma, Research and Early Development, Roche Innovation Center Shanghai, Shanghai, China; ^4^ Department of Pathology, Georgetown University Medical Center, Washington, DC, United States

**Keywords:** chronic hepatitis B, woodchuck, TLR7 agonism, RG7854, functional cure, entecavir, innate immune response, adaptive immune response

## Abstract

Conventional treatment of chronic hepatitis B (CHB) is rarely curative due to the immunotolerant status of patients. RG7854 is an oral double prodrug of a toll-like receptor 7 (TLR7) agonist that is developed for the treatment of CHB. The therapeutic efficacy, host immune response, and safety of RG7854 were evaluated in the woodchuck model of CHB. Monotreatment with the two highest RG7854 doses and combination treatment with the highest RG7854 dose and entecavir (ETV) suppressed viral replication, led to loss of viral antigens, and induced seroconversion in responder woodchucks. Since viral suppression and high-titer antibodies persisted after treatment ended, this suggested that a sustained antiviral response (SVR) was induced by RG7854 in a subset of animals. The SVR rate, however, was comparable between both treatment regimens, suggesting that the addition of ETV did not enhance the therapeutic efficacy of RG7854 although it augmented the proliferation of blood cells in response to viral antigens and magnitude of antibody titers. The induction of interferon-stimulated genes in blood by RG7854/ETV combination treatment demonstrated on-target activation of TLR7. Together with the virus-specific blood cell proliferation and the transient elevations in liver enzymes and inflammation, this suggested that cytokine-mediated non-cytolytic and T-cell mediated cytolytic mechanisms contributed to the SVR, in addition to the virus-neutralizing effects by antibody-producing plasma cells. Both RG7854 regimens were not associated with treatment-limiting adverse effects but accompanied by dose-dependent, transient neutropenia and thrombocytopenia. The study concluded that finite, oral RG7854 treatment can induce a SVR in woodchucks that is based on the retrieval of antiviral innate and adaptive immune responses. This supports future investigation of the TLR7 agonist as an immunotherapeutic approach for achieving functional cure in patients with CHB.

## Introduction

Chronic infection with hepatitis B virus (HBV) affects approximately 296 million individuals worldwide and results in 820,000 deaths every year due to HBV-associated liver disease, making this viral infection one of the most serious global health issues (1). Carriers of HBV have a high risk of developing chronic hepatitis B (CHB), liver cirrhosis, and hepatocellular carcinoma (HCC) and will die without therapeutic intervention and/or liver transplantation. The hallmarks of CHB are high levels of viremia (HBV DNA) and surface antigenemia (HBsAg) in the circulation, while antibodies to HBsAg (anti-HBs antibodies) are characteristically absent (2, 3). Several studies have shown the importance of these viral markers in the HBV-related disease outcome. Loss of HBsAg either mediated by antiviral treatment or induced spontaneously is associated with a lower risk of liver disease progression to HCC (4, 5), while development of anti-HBs antibodies after prophylactic vaccination or resolution of acute HBV infection offers lifelong immunity (6, 7). However, the currently approved drugs, including oral nucleos(t)ide analogues (NAs) and systemic (pegylated) interferon-alpha (IFN-α), rarely achieve immunological control of HBV or a functional cure, which is defined as sustained suppression of HBV DNA and loss of HBsAg after treatment discontinuation, with or without seroconversion to anti-HBs antibodies (8). The underlying reason is that NAs effectively suppress HBV DNA synthesis and reduce liver inflammation but require lifelong administration, since these direct-acting antivirals do not affect the persistent covalently-closed circular (ccc) viral DNA genome within the nucleus of infected hepatocytes, and viral relapse is typically observed after treatment cessation (8). IFN-α directly targets HBV cccDNA and suppresses its functions (9–12) and induces an antiviral immune response in patients, but is sometimes associated with severe side effects (8). The HBV cure rate accomplished with IFN-α is slightly higher than with NAs, and combination treatment with both drugs increases this rate to approximately 10% of patients (8, 13). Thus, novel therapeutics are urgently needed for use as single agents or for incorporation into already applied treatment regimens, with the overall goal to achieve HBV functional cure in a majority of patients after a finite course of treatment.

CHB in patients is associated with insufficient innate and adaptive immunity against HBV (3, 14–18). Unlike many other viruses, HBV avoids the induction of a type-I IFN-based host innate immune response during initial establishment of the infection, and thus displays a stealth-like behavior (19). During progression to chronic HBV infection, the high levels of viral proteins in the periphery and liver are thought to interfere with the pathway activation of pathogen recognition receptors (PRRs) (17, 20–24). Viral proteins further modulate innate immune cell subsets (25–27) although the altered function of dendritic cells (DCs) appears to correlate more with liver disease progression than with antigen load (28). Prolonged exposure to viral proteins rather than high antigen load during chronic HBV infection is further believed to be responsible for the functional impairment of HBV-specific T-cells and HBsAg-specific B-cells (29–32). These immunodeficiencies have shifted the focus of anti-HBV drug discovery to immunomodulation as a therapeutic strategy for reviving the impaired antiviral immunity in patients with CHB (8).

Since HBV is not actively or entirely inhibiting the function of PRRs (33, 34), small molecules stimulating selected receptors have been developed and several agonists were evaluated first in animal models of HBV and subsequently in patients (35, 36). Among these, agonists of toll-like receptor 7 (TLR7) appear promising therapeutics that may be able to overcome the HBV-associated immunodeficiencies present in patients. TLR7 is predominately expressed within the endosome of antigen presenting cells (APCs), including plasmacytoid (p) DCs and B-lymphocytes, and naturally recognizes viral single-stranded RNA (37). Following receptor activation, the downstream signaling cascade leads to the production of multiple type-I IFN isotypes and T-cell attractant chemokines, enhancement of antigen processing and presentation by APCs, and upregulation of costimulatory molecules critical for the cross-priming of cytotoxic T-cells (38, 39), all of which could be beneficial in restoring innate and adaptive immunity for subsequent HBV control. GS-9620, the first in-class oral TLR7 agonist developed for the treatment of CHB produced a long-lasting viral suppression in chimpanzees infected with HBV (40) and a sustained antiviral response (SVR) or functional cure in a subset of woodchucks infected with woodchuck hepatitis virus (WHV) (41). The unprecedented antiviral effect achieved in the latter animal model of HBV with any single agent therapy evaluated so far was due to an additional activation of woodchuck TLR8 by high GS-9620 dosage (42). However, GS-9620 treatment of patients failed to mediate therapeutic efficacy at tolerated doses when used alone or in combination with a NA (43, 44), but improved the responses of HBV-specific natural killer (NK) cells and T-cells (45). APR002, another oral TLR7 agonist, induced a functional cure in a subset of woodchucks, but only when administered together with the nucleoside analogue entecavir (ETV) (46).

The Eastern woodchuck (*Marmota monax*), chronically infected with WHV, is an established, immunocompetent animal model for studies of HBV pathogenesis and therapy. Like HBV, WHV is a member of the genus Orthohepadnavirus, and both viruses are closely related regarding their genome structure and replication mechanism (47). Host immune response to WHV and virus-induced liver disease progression in woodchucks parallel HBV infection in humans (35, 48–52). Woodchucks are applied in the assessment of the safety and therapeutic efficacy of new drugs developed for the treatment of CHB and HCC, and the preclinical use of this model is predictive of antiviral efficacy of NAs (53, 54) and immunomodulators against HBV in patients (42, 55).

We report here the evaluation of RG7854, an oral double prodrug of a TLR7-specific agonist developed by F. Hoffmann-La Roche, Ltd., in woodchucks with CHB. RG7854 is converted *in vivo* to its active metabolite RO7011785 *via* hydrolysis by mainly carboxylesterase 2 and oxidation by aldehyde oxidase (56). The initial dose-finding study in woodchucks assessed tolerability and potency of three increasing RG7854 doses. Since the safe and potent NAs are expected to remain the pillar of any future anti-HBV therapy, the subsequent combination treatment study in woodchucks assessed the antiviral benefit of high RG7854 dosage, when administered together with ETV. Like RG7854 monotreatment, RG7854/ETV combination treatment resulted in undetectable viral DNA, loss of WHV surface (WHsAg) and e antigens (WHeAg), and seroconversion to antibodies against both viral proteins (anti-WHs and anti-WHe antibodies) in a subset of woodchucks that was characterized by remarkably high titers of virus-neutralizing antibodies, but did not further enhance the rate of functional cure beyond that of RG7854 alone.

## Materials and Methods

### Investigational Drugs

RG7854 and ETV were manufactured by F. Hoffmann-La Roche and provided as a dry powder. RG7854 was dissolved in vehicle (i.e., 2% (w/v) Klucel LF (hydroxypropylcellulose), 0.09% (w/v) methylparaben, and 0.01% (w/v) propylparaben in water). ETV was also dissolved in vehicle (i.e., ultrapure water). Drugs were mixed with woodchuck diet (Dyets, Inc., Bethlehem, PA) and orally administered to animals within 30 minutes after preparation using an aluminum luer lock tube with gavage needle. Control animals were administered placebo (i.e., vehicle) mixed with woodchuck diet.

### Study Design

**Figure 1:**
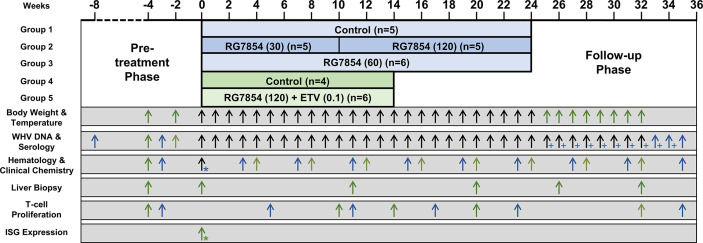
Study design. In the monotreatment study (_▀_), woodchucks were dosed with vehicle or RG7854 (30/120 or 60 mg/kg orally, QOD) for 24 weeks and followed for another 11 weeks. Starting in week 10 of treatment, the 30 mg/kg dose was increased to 120 mg/kg in Group 2 and administered for 14 weeks. In the combination treatment study (_▀_), woodchucks were dosed with vehicle or RG7854 (120 mg/kg orally, QOD) together with ETV (0.1 mg/kg orally, once daily) for 14 weeks and followed for another 18 weeks. Arrows indicate the time of measurements for the specific parameters listed. Black arrows indicate parameters measured in both studies. * Pre-and post-dose samples were collected. ^+^ Only serum WHV DNA was measured.

Woodchucks received humane care according to the criteria outlined in the Guide for the Care and Use of Laboratory Animals. Animal protocols including woodchucks were approved by the Institutional Animal Care and Use Committee of Northeastern Wildlife, Inc. (Harrison, ID) and Georgetown University (Washington, DC). All animals were born in captivity at the animal facilities of Northeastern Wildlife, Inc., infected with WHV at three days of age to model vertical HBV transmission in humans, and raised to adulthood prior to use in the RG7854 mono and RG7854/ETV combination treatment studies. Before study initiation, chronic WHV carrier woodchucks of both genders were confirmed positive for serum WHV DNA, WHsAg, and WHeAg, and negative for anti-WHs and anti-WHe antibodies. Woodchucks were allocated to three and two groups in the mono or combination treatment studies, respectively (**Figure 1**; **Supplementary Table 1**), and randomized within blocks (i.e., sex) and factors (i.e., body weight). If needed, animals were moved between the groups based on other parameters, including pretreatment serum WHV DNA and WHsAg loads and liver enzyme levels, for achieving comparable ranges within each group. Animal research staff was not blinded in regard to treatment administration and animal procedures. However, laboratory research staff was blinded to animal group/treatment allocation during sample processing and analysis. Woodchucks undergoing monotreatment were orally treated every other day (QOD) for 24 weeks with vehicle (Group 1; n=5) or RG7854 (30/120 or 60 mg/kg) (Group 2; n=5 or Group 3; n=6) and then followed for additional 11 weeks until the end of the study (EOS) at week 35. The RG7854 starting doses of 30 and 60 mg/kg were selected to match the proportional increase in plasma exposure of the active form of the TLR7 agonist RO7011785 in WHV-naïve woodchucks after administration of single, oral doses ranging from 3 to 30 mg/kg (data not shown). Compared to the efficacious range of RG7854 determined previously in an HBV mouse model (57), both starting doses represented a targeted 2-3-fold higher plasma exposure in chronic WHV carrier woodchucks to account for differences in metabolic size (58). However, due to the absence of immediate antiviral effects after treatment initiation, the RG7854 dose in Group 2 was increased from 30 to 120 mg/kg starting in week 10 and continuing for additional 14 weeks of treatment. Woodchucks undergoing combination treatment were orally treated QOD for 14 weeks with vehicle (Group 4; n= 4) or RG7854 (120 mg/kg) together with daily ETV (0.1 mg/kg) (Group 5; n=6) and then followed for additional 18 weeks until the EOS at week 32. Thus, woodchucks of Groups 2 and 5 underwent high dose (120 mg/kg) RG7854 mono or combination treatment for 14 weeks and animals of Group 3 received intermediate dose (60 mg/kg) RG7854 monotreatment for 24 weeks. A control group undergoing ETV monotreatment was not included in this study due to the paucity of woodchucks with chronic WHV infection.

### Drug Safety and Mortality

Clinical observations were made daily, while measurements of body weight and temperature were obtained weekly. Hematology and clinical chemistry markers were determined at regular intervals. Mortality associated with RG7854 and ETV was not observed. In the monotreatment study, woodchucks F7991 (Group 1), F7996 (Group 2), and F8226 (Group 3) were euthanized during treatment in weeks 18, 7, or 17, respectively. Woodchuck F7934 (Group 1) was found dead and M7979 (Group 2) was euthanized during the follow-up in weeks 25 or 26, respectively. Scheduled euthanasia or death were due to the development of end-stage HCC in all cases. In the combination treatment study, woodchuck F5008 died due to internal hemorrhage after the liver biopsy procedure in week 11.

### Animals and Procedures

Woodchucks were pair-housed in stainless-steel cages with solid floors and aspen contact bedding. Animals received aspen woodblocks for enrichment. The temperature was maintained at 65 to 70°F (approximately 18 to 21°C) and lights were on a 12/12-hour cycle. Woodchucks were fed laboratory chow formulated and specifically pelleted for woodchucks (Dyets) and had access to tap water *ad libitum*. Woodchucks were not fasted for any procedure and all procedures were conducted during the light cycle. Procedures involving body weight and temperature measurements, blood collection, liver biopsy, liver ultrasonography, and euthanasia were performed under isoflurane inhalation and/or ketamine/xylazine intramuscular injection anesthesia. Blood samples for testing serology, hematology, and clinical chemistry were obtained *via* femoral venipuncture. Liver tissues for assessing WHV nucleic acids and histology were collected by ultrasound-guided, percutaneous liver biopsy. Blood and liver tissues were always obtained prior to drug and vehicle administration.

### Serum WHV Markers

Serum WHV DNA load was assayed quantitatively by slot-blot hybridization and PCR (lower limit of detection (LLOD): 600 WHV genomic equivalents (ge) or copy numbers per mL serum), as described previously (58, 59). Serum WHsAg load was assayed quantitatively by ELISA (LLOD: 5 ng WHsAg/mL serum) comparable to the assay described previously (59, 60). Serum WHeAg load was assayed qualitatively using a cross-reactive ELISA (DiaSorin, Minneapolis, MN) by following the manufacturer’s protocol. Results were obtained as an optical density read out, and a value of ≤0.060 optical density units (ODU) indicated absence of WHeAg. Serum anti-WHs antibodies were assayed quantitatively using an established enzyme immunoassay (LLOD: 100 standard units (StdU)/mL serum), as described previously (59, 60). Serum anti-WHe antibodies were assayed qualitatively using a cross-reactive ELISA (DiaSorin) by following the manufacturer’s protocol. An ODU value of ≥2.33 (i.e., sample ODU value at pretreatment (T0) minus sample ODU value in a given study week) indicated presence of anti-WHe antibodies.

### Liver WHV Markers

Intrahepatic levels of WHV DNA replicative intermediates (RI) and cccDNA were assayed quantitatively by Southern blot hybridization, while intrahepatic WHV RNA levels, consisting of pre-genomic and surface RNA molecules, were determined quantitatively by Northern blot hybridization, as described previously (58, 59). Woodchuck β-actin was used for the normalization of WHV nucleic acid concentrations. Both hybridization assays provided results spanning up to >1 and >2 orders of magnitude of detection for WHV RNA and WHV cccDNA or WHV DNA RI molecules, respectively (LLOD: 2 pg WHV DNA or WHV RNA/µg cellular nucleic acids).

### Hematology and Clinical Chemistry Markers

Blood samples for hematology and serum clinical chemistry were analyzed at the Animal Health Diagnostic Center of Cornell University (Ithaca, NY) using parameters established for woodchucks (61, 62). Hematology markers included white blood cells, segmented neutrophils, banded neutrophils, lymphocytes, monocytes, eosinophils, basophils, red blood cells, hemoglobin, hematocrit, mean cell volume, mean cell hemoglobin, mean cell hemoglobin concentration, red cell distribution width, platelet count, and mean platelet volume. Clinical chemistry markers included alkaline phosphatase, alanine aminotransferase (ALT), aspartate aminotransferase (AST), gamma-glutamyl transferase (GGT), sorbitol dehydrogenase (SDH), sodium, potassium, chloride, bicarbonate, anion gap, sodium/potassium ratio, urea, creatinine, calcium, phosphate, magnesium, total protein, albumin, globulin, albumin/globulin ratio, glucose, total bilirubin, direct bilirubin, indirect bilirubin, amylase, cholesterol, creatine kinase, iron, total iron binding capacity, percent saturation, lipemia, hemolysis, and icterus.

### Histology

Paraffin-embedded liver tissues were sectioned (5 microns) and stained with hematoxylin and eosin at the Histopathology & Tissue Shared Resource (HTSR) Laboratory of Georgetown University (Washington, DC). Tissue sections were examined by a board-certified pathologist (BVK). Liver disease progression, including portal and sinusoidal hepatitis, bile duct proliferation, steatosis, fibrosis, and necrosis, was assessed *via* criteria developed for woodchuck liver (63, 64), as well as by using the METAVIR scale for scoring human liver.

### T-Cell Proliferation

Peripheral blood mononuclear cells (PBMCs) were isolated from whole blood by Ficoll-Paque density gradient centrifugation and cultured in AIM-V medium (Invitrogen/Thermo Fisher Scientific, Waltham, MA) in 96-well opaque plates (Sigma, St. Louis, MO), as described previously (65). PBMCs were stimulated with 0.02% (v/v) DMSO (Sigma, unstimulated medium control), 0.5 µg/mL lipopolysaccharide (LPS; Sigma, no-peptide control), and pools of peptides covering the entire WHV core antigen (WHcAg) or WHsAg (Invitrogen/Thermo Fisher Scientific). Peptides were dissolved in sterile saline for obtaining a final concentration of 10.0 µg/mL of each peptide in 0.02% (v/v) DMSO. T-cell proliferation was determined after five days with the CellTiter Glo One Solution assay (Promega, Madison, WI) by following the manufacturer’s protocol. The derived luminescence signal of triplicate cultures was averaged and expressed as a fold-change by dividing the average signal in the presence of stimulator (LPS or WHcAg- or WHsAg-derived peptides) by that in the absence of stimulator (DMSO-containing medium). Results were further represented as a fold-change relative to the pretreatment baseline. A fold-change of ≥2.1 was considered a positive result for WHV-specific T-cell proliferation (66).

### IFN-Stimulated Gene Induction

The induction of IFN-stimulated genes (ISGs) in blood was determined by using reverse transcription PCR and woodchuck-specific primers and probes (**Supplementary Table 2**), as described previously (55, 66). In brief, total RNA from whole blood collected in PAXgene blood tubes (Qiagen, Redwood City, CA) was isolated using the PAXgene Blood miRNA kit (Qiagen) with on-column DNase I digestion using RNase-free DNase by following the manufacturer’s protocol. Messenger RNA in these samples was then reverse transcribed using oligo(dT) and the High-Capacity cDNA Reverse Transcription kit (Applied Biosystems, Foster City, CA). Expression changes of IFN-induced 17 kDa protein (*ISG15*), IFN-induced guanosine triphosphate-binding protein (*MX1*), 2’-5’-oligoadenylate synthetase 1 (*OAS1*), and IFN-γ induced protein 10 (*CXCL10* or IP-10) were determined on an ABI 7500 Real Time PCR System instrument (Applied Biosystems) by using the TaqMan Gene Expression Master mix (Applied Biosystems). Woodchuck 18S ribosomal RNA expression was used to normalize target gene expression. Transcript levels of ISGs were calculated as a fold-change relative to the pretreatment baseline level using the formula 2^-Δ^ *^Ct^*. A fold-change of ≥2.1 was considered a positive result for increased transcription (66).

### Statistical Analysis

All experimental data was carefully inspected for consistency and completeness before statistical analysis. Values below detectable concentration or assay limit were replaced by either the minimum of all measured values (4 and 3 international units (IU)/L for serum ALT or GGT, respectively) or by the corresponding assay LLOD (600 ge/mL for serum WHV DNA, 5 ng/mL for serum WHsAg, 2 pg/µg for intrahepatic WHV DNA RI, cccDNA, and RNA). Data for serum WHV DNA, WHsAg, and anti-WHs antibodies were transformed to a log_10_ scale and arithmetically averaged prior to statistical analysis. Whenever appropriate, mean parameters (i.e., body weight and temperature, hematology, clinical chemistry, serum and liver WHV markers, blood host markers, and liver pathology) at each timepoint of the study were compared to the values at pretreatment and/or between the three or two groups undergoing RG7854 mono or RG7854/ETV combination treatment, respectively, using an unpaired Student’s *t*-test with equal variance. *P*< 0.05 was considered statistically significant. Sex was not considered a factor in the statistical analysis.

## Results

### RG7854 Treatment, Alone and Together With ETV, Induced Suppression of Serum Viremia and Antigenemia and Seroconversion in a Subset of Woodchucks

**Figure 2:**
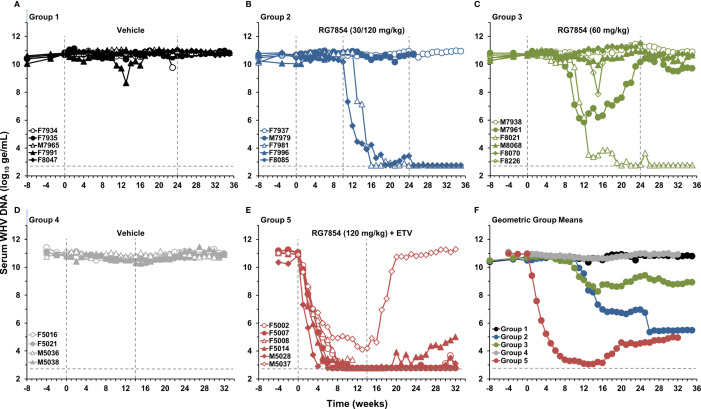
Effect of RG7854, alone and together with ETV, on serum viremia levels. Kinetics of WHV DNA load in individual woodchucks administered **(A)** placebo or RG7854 at doses of **(B)** 30/120 mg/kg or **(C)** 60 mg/kg in the monotreatment study and **(D)** placebo or **(E)** RG7854 at a dose of 120 mg/kg plus ETV in the combination treatment study. **(F)** Geometric group mean WHV DNA loads. The outer vertical dotted lines represent the duration of 24-week monotreatment or 14-week combination treatment, while the inner vertical dotted line represents the switch from 30 to 120 mg/kg RG7854 in Group 2 during week 10 in this and the following figures. The horizontal dotted lines indicate the detection limit for WHV DNA by quantitative polymerase chain reaction (i.e., 600 ge/mL). The geometric mean WHV DNA load in Group 3 was significantly reduced compared to Group 1 at T0 and at week 7 (*P*< 0.05) (Student’s *t*-test). Compared to Group 1, the geometric mean WHV DNA load in Group 2 was not significantly different (*P*> 0.05). The geometric mean WHV DNA load in Group 5 was significantly reduced compared to Group 4 during weeks 1-32 (*P*< 0.05). ge, genome equivalents or copy numbers.

**Figure 3:**
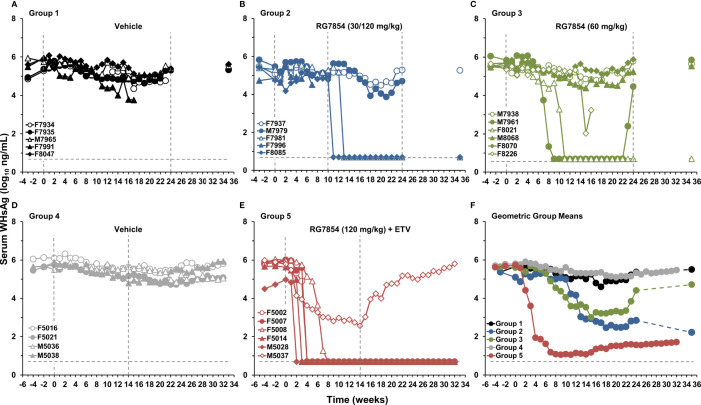
Effect of RG7854, alone and together with ETV, on serum surface antigenemia levels. Kinetics of WHsAg load in individual woodchucks administered **(A)** placebo or RG7854 at doses of **(B)** 30/120 mg/kg or **(C)** 60 mg/kg in the monotreatment study and **(D)** placebo or **(E)** RG7854 at a dose of 120 mg/kg plus ETV in the combination treatment study. **(F)** Geometric group mean WHsAg loads. The horizontal dotted lines indicate the detection limit for WHsAg by quantitative enzyme-linked immunosorbent assay (i.e., 5 ng/mL). The geometric mean WHsAg loads in Group 2 and Group 3 were significantly reduced compared to Group 1 at T0 and at weeks 1, 6, 7, and 8 or at week 15, respectively (*P*< 0.05) (Student’s *t*-test). Compared to Group 4, the geometric mean WHsAg load in Group 5 was significantly reduced during weeks 4-32 (*P*< 0.05).

**Figure 4:**
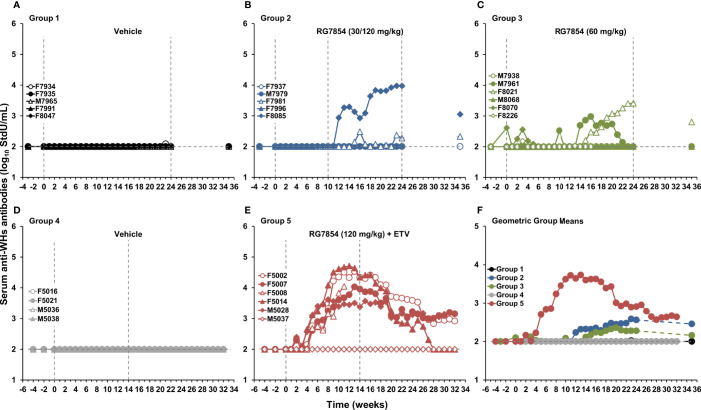
Effect of RG7854 treatment, alone and together with ETV, on the elicitation of serum antibodies to WHsAg. Kinetics of anti-WHs antibody titer in individual woodchucks administered **(A)** placebo or RG7854 at doses of **(B)** 30/120 mg/kg or **(C)** 60 mg/kg in the monotreatment study and **(D)** placebo or **(E)** RG7854 at a dose of 120 mg/kg plus ETV in the combination treatment study. **(F)** Geometric group mean anti-WHs antibody titers. The horizontal dotted lines indicate the detection limit for anti-WHs antibodies by quantitative enzyme immunoassay (i.e., 100 StdU/mL). The geometric mean anti-WHs antibody titers in Group 2 and Group 3 were not significantly different to Group 1 (*P*> 0.05) (Student’s *t*-test). Compared to Group 4, the geometric mean anti-WHs antibody titer in Group 5 was significantly increased during weeks 5-27 (*P*< 0.05). StdU, standard units.

The antiviral efficacy of RG7854 was first evaluated in a dose-finding study in woodchucks with established chronic WHV infection (**Figure 1**). Sixteen woodchucks were assigned to repeat-dose monotreatment with either vehicle (Group 1; n=5) or RG7854 at doses of 30 mg/kg (Group 2; n=5) and 60 mg/kg (Group 3; n=6) for 24 weeks. Since an interim analysis indicated that both RG7854 doses did not induce marked declines in serum WHV markers or elicited antibodies in woodchucks immediately after treatment initiation (**Figures 2**–**4**; **Supplementary Figures 1, 2**), the dose in Group 2 was increased from 30 to 120 mg/kg in week 10 and administered for 14 weeks, while the original 60 mg/kg dose in Group 3 continued for 24 weeks.

In contrast to low dose (30 mg/kg) RG7854, the switch to high dose (120 mg/kg) RG7854 treatment produced a rapid decline in serum WHV DNA, WHsAg, and WHeAg in two of four woodchucks in Group 2 (i.e., F7981 and F8085) within 1-6 weeks (**Figures 2**-**4**; **Supplementary Figures 1, 2**). Both animals achieved a marked reduction in viremia and complete loss of detectable surface and e antigenemia, which was associated with emerging anti-WHs but not anti-WHe antibodies during treatment, and with a notably high anti-WHs antibody titer in F8085 that waned thereafter. The response to intermediate dose (60 mg/kg) RG7854 treatment in Group 3 was more varied, with two of five woodchucks displaying a more pronounced antiviral effect, starting 6-10 weeks after treatment initiation. F8021 achieved complete suppression of WHV DNA and loss of WHsAg and WHeAg, and elicited antibodies to both antigens during treatment. M7961 had transient reductions in WHV DNA and WHsAg, with minor changes in WHeAg and a transient induction of anti-WHs but not of anti-WHe antibodies, and experienced a gradual viral rebound towards the EOS that already started during treatment. A comparable antiviral response was not noted for other woodchucks in Groups 2 and 3, and marked changes in WHV markers and antibodies were absent in control animals of Group 1. Compared to Group 1, the declines in serum WHV DNA, WHsAg, and WHeAg loads and the increases in anti-WHs antibody titer and anti-WHe antibody level in Groups 2 and 3 were not significant during most of the study.

Since therapeutic efficacy was observed shortly after the switch to the high RG7854 dose, treatment with the TLR7 agonist at this dose in combination with ETV was subsequently tested in chronic WHV carrier woodchucks (**Figure 1**). Combination treatment with ETV was selected because most therapeutic interventions based on immunomodulation will likely be provided concurrently or as add-on to standard-of-care treatment with NAs. Thus, ten woodchucks were assigned to repeat-dose treatment with either vehicle (Group 4; n=4) or RG7854 (120 mg/kg) together with ETV (0.1 mg/kg) for 14 weeks (Group 5; n=6). The ETV dosage was selected based on another reported study in woodchucks (46).

Similar to high dose (120 mg/kg) RG7854 monotreatment, RG7854/ETV combination treatment produced rapid and marked declines in serum WHV DNA, WHsAg, and WHeAg within 1-5 weeks after initiation in four of five woodchucks in Group 5 (**Figures 2**, **3**; **Supplementary Figure 1**). F5002, F5007, and M5028 accomplished sustained suppression of viremia and loss of surface and e antigenemia, and elicited anti-WHs and anti-WHe antibodies immediately thereafter which persisted throughout the study (**Figure 4**; **Supplementary Figure 2**). Seroconversion was again associated with remarkable high levels of anti-WHs antibodies although titers started to wane at the end of treatment and more so during the follow-up. Anti-WHe levels waned as well but the decline was more gradual. F5014 also showed marked reductions in WHV DNA and WHeAg, loss of WHsAg, and a transient induction of anti-WHs but not of anti-WHe antibodies; however, this woodchuck experienced a relapse in viremia towards the EOS after treatment cessation. The treatment response in these four animals was clearly different to the less pronounced and always transient declines in viremia and surface and e antigenemia noted for M5037, with absent antibody response to both antigens. Comparable changes in viral markers and antibodies were not present in control animals of Group 4, and the declines in serum WHV DNA, WHsAg, and WHeAg loads and the increase in anti-WHs antibody titer in woodchucks of Group 5 were significant during most of the study.

### RG7854 Mono and Combination Treatment Resulted in a SVR in a Comparable Number of Woodchucks

Based on the above observations and for correlative analyses of the RG7854 mono and RG7854/ETV combination treatment responses, response groups were defined as the following: Responders (R) had serum WHV DNA <10^3^ genomic equivalents (ge)/mL, WHsAg ≤5 ng/mL, and anti-WHs antibodies >10^3^ standard units (StdU)/mL at the end of treatment. Non-Responders (NR) had minimum WHV DNA >10^8^ ge/mL, minimum WHsAg >10^3^ ng/mL, and absent anti-WHs antibodies (≤100 StdU/mL) at the end of treatment. Partial Responders (PR) had WHV DNA and WHsAg loads between Responders and Non-Responders and anti-WHs antibody titers >100 but <10^3^ StdU/mL at the end of treatment (**Table 1**). This rather stringent definition revealed that F8021 of Group 3 was a Responder to 24-week intermediate RG7854 dose monotreatment, while F7981 and F8085 of Group 2 and M7961 of Group 3 were Partial Responders to 14-week high or 24-week intermediate RG7854 dose monotreatment, respectively. All other animals in both groups were Non-Responders. This definition further revealed that F5002, F5007, F5014, and M5028 of Group 5 were Responders to 14-week high RG7854 dose/ETV combination treatment, while M5037 was a Non-Responder.

**Table 1:** Correlative analyses of RG7854 mono and RG7854/ETV combination treatment responses.

Treatment Group	Treatment	Animal Identification	Treatment Response Group^a^	Sustained Viral Response^b^	Baseline/Max. Decline Serum WHV DNA (log_10_ ge/mL)^c^	Baseline/Max. Decline Serum WHsAg (log_10_ ng/mL)^c^	Baseline/Max. Increase Serum Anti-WHs Antibodies(log_10_ StdU/mL)^d^
1	Vehicle	F7934	ND^††^		10.68/0.91	5.28/0.93	2.00/0.09
		F7935	NR	–	10.83/0.48	5.37/0.74	2.00/0.00
		M7965	NR	–	10.80/0.06	5.76/0.84	2.00/0.00
		F7991	ND^†^		10.73/2.06	5.96/2.20	2.00/0.00
		F8047	NR	–	10.76/0.15	5.93/0.99	2.00/0.00
2	RG7854 (30/120 mg/kg)	F7937	NR	–	10.60/0.10	5.08/0.60	2.00/0.00
		M7979	NR^††^		10.66/0.51	5.47/1.61	2.00/0.00
		F7981	PR	+	10.70/7.92^e^	5.32/4.62^f^	2.00/0.49
		F7996	ND^†^		10.03/0.00	4.85/0.33	2.00/0.00
		F8085	PR	+	10.45/7.67^e^	4.75/4.05^f^	2.00/1.98
3	RG7854 (60 mg/kg)	M7938	NR	–	10.67/0.07	5.42/0.63	2.00/0.00
		M7961	PR	–	10.73/4.87	5.84/5.14^f^	2.00/0.98
		F8021	R	+	10.63/7.85^e^	5.69/4.99^f^	2.00/1.41
		M8068	NR	–	10.68/0.72	5.57/1.18	2.00/0.00
		F8070	NR	–	10.70/0.28	5.72/0.77	2.61/-0.05
		F8226	ND^†^		10.70/2.83	5.36/3.33	2.00/0.18
4	Vehicle	F5016	NR	–	11.06/0.55	6.10/0.74	2.00/0.00
		F5021	NR	–	10.89/0.45	5.75/1.03	2.00/0.00
		M5036	NR	–	10.99/0.31	5.50/0.65	2.00/0.00
		M5038	NR	–	10.79/0.51	5.86/1.07	2.00/0.00
5	RG7854 (120 mg/kg) + ETV (0.1 mg/kg)	F5002	R	+	11.04/8.26^e^	5.97/5.28^f^	2.00/2.53
		F5007	R	+	11.07/8.29^e^	6.01/5.31^f^	2.00/2.03
		F5008	ND^†^		11.00/8.08	5.97/5.28^f^	2.00/2.05
		F5014	R	–	10.96/8.19^e^	5.63/4.93^f^	2.00/2.71
		M5028	R	+	10.83/8.05^e^	4.99/4.29^f^	2.00/1.62
		M5037	NR	–	10.91/6.80	5.79/3.22	2.00/0.00

a Treatment response groups were defined as follows: R, Responders, serum WHV DNA <10^3^ ge/mL, serum WHsAg ≤5 ng/mL, and anti-WHs antibodies >10^3^ StdU/mL at the end of treatment; NR, Non-Responders, minimum serum WHV DNA >10^8^ ge/mL, minimum serum WHsAg >10^3^ ng/mL, and absent anti-WHs antibodies (≤100 StdU/mL) at the end of treatment; PR, Partial Responders, serum WHV DNA and WHsAg loads between Responders and Non-Responders and anti-WHs antibody titer >100 but <10^3^ StdU/mL at the end of treatment.

b Sustained viral response was defined as serum WHV DNA <10^3^ ge/mL, serum WHsAg ≤5 ng/mL, and anti-WHs antibodies present at the EOS.

c The maximum reductions in serum WHV DNA and WHsAg during treatment and/or follow-up were calculated relative to the week 0 (T0) timepoint (pretreatment baseline).

d The maximum increase in serum anti-WHs antibodies during treatment and/or follow-up was calculated relative to the week 0 (T0) timepoint (pretreatment baseline).

e Viremia in animals F7981, F8021, F8085, F5002, F5007, F5014, and M5028 was < lower limit of detection (LLOD; 600 ge/mL) at one or more timepoints; the LLOD was used to calculate the maximum WHV DNA decline in these animals.

f Antigenemia in animals M7961, F7981, F8021, F8085, F5002, F5007, F5008, F5014, and M5028 was < LLOD (5 ng/mL) at one or more timepoints; the LLOD was used to calculate the maximum WHsAg decline in these animals.

^†^Treatment response group was not determined (ND) as animal died during treatment: F7991 (Group 1), F7996 (Group 2), and F8226 (Group 3) were euthanized in weeks 18, 7, or 17, respectively, due to symptoms associated with end-stage HCC. F5008 (Group 5) died in week 11 due to liver biopsy-related hemorrhage.

^††^Treatment response group was ND as animal died during the follow-up: F7934 (Group 1) was found dead in week 25, cause of death was attributed to terminal HCC. M7979 (Group 2) was euthanized in week 26 due to symptoms associated with end-stage HCC.

For a further delineation of the RG7854 mono and combination treatment responses in regard to durability, a SVR was defined as serum WHV DNA <10^3^ ge/mL, WHsAg ≤5 ng/mL, and anti-WHs antibodies present at the EOS (**Table 1**). Based on WHV DNA assayed between the end of monotreatment and the EOS and WHsAg and anti-WHs antibodies measured at the EOS in woodchucks of Groups 1-3, this suggested that a SVR was achieved in F7981, F8021, and F8085 of Groups 2 and 3 at the end of the study in week 35. Based on the viremia, antigenemia, and antibody data obtained between the end of combination treatment and the EOS, this further indicated that a SVR was accomplished in F5002, F5007, and M5028 of Group 5 at the end of the study in week 32. Thus, 1 out of 5 surviving woodchucks in Group 3 (20%), 2 out of 3 surviving animals in Group 2 (67%), and 3 out of 5 surviving animals in Group 5 (60%) achieved a SVR. Although a trend towards higher SVR percentage for combination over mono treatment (38% vs. 60%) may exist, the comparable percentage between animals receiving the high RG7854 dose during mono and combination treatment (67% vs. 60%) and the equal number of 3 woodchucks with SVR each in the mono and combination treatment regimens suggested no apparent added benefit of ETV in regard to the observed therapeutic efficacy.

### RG7854/ETV Combination Treatment Produced Sustained Suppression of Viral Replication in the Liver of Woodchucks With SVR

**Figure 5:**
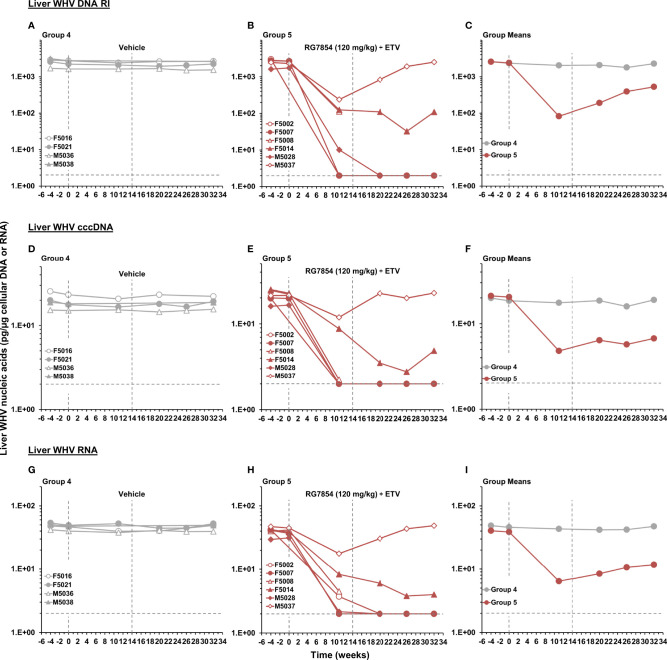
Effect of RG7854 together with ETV on liver viremia levels. Kinetics of WHV DNA RI, cccDNA, and RNA loads in individual woodchucks administered **(A, D, G)** placebo or **(B, E, H)** RG7854 at a dose of 120 mg/kg plus ETV in the combination treatment study. **(C, F, I)** Group mean WHV nucleic acids. The vertical dotted lines represent the duration of 14-week combination treatment. The horizontal dotted lines indicate the detection limit for WHV nucleic acids by quantitative Southern and Northern blot hybridization (i.e., 2 pg/µg cellular DNA or RNA). The mean WHV DNA RI, cccDNA, and RNA loads in Group 5 were significantly reduced compared to Group 4 at weeks 11, 20 and 32, at weeks 11 and 32, or at weeks 11, 20, and 32, respectively (*P*< 0.05) (Student’s *t*-test).

For further confirming the SVR mediated by RG7854/ETV combination treatment, changes in intrahepatic WHV nucleic acids were assayed in sequential liver biopsies obtained during the study (**Figure 5**). Consistent with the effects on serum viremia and antigenemia, Responders in Group 5 had markedly reduced WHV DNA RI, cccDNA, and RNA loads in liver as early as week 11 of treatment. All WHV nucleic acids became undetectable six weeks after drug withdrawal in F5002, F5007, and M5028 with SVR, and stayed absent until the EOS. F5014, a Responder without SVR, experienced reductions in these viral markers during and following treatment as well, but the declines were more gradual and WHV nucleic acids relapsed at the EOS, as also observed for serum viremia. M5037, a Non-Responder, had the least decline in viral markers during treatment and WHV nucleic acids returned to baseline level after treatment cessation. Control animals in Group 4 had no comparable changes in intrahepatic WHV markers, and the declines in WHV DNA RI, cccDNA, and RNA loads in woodchucks of Group 5 were significant during most of the study.

### The First Dose of RG7854/ETV Combination Treatment Induced ISGs in the Periphery of Woodchucks With SVR

**Figure 6:**
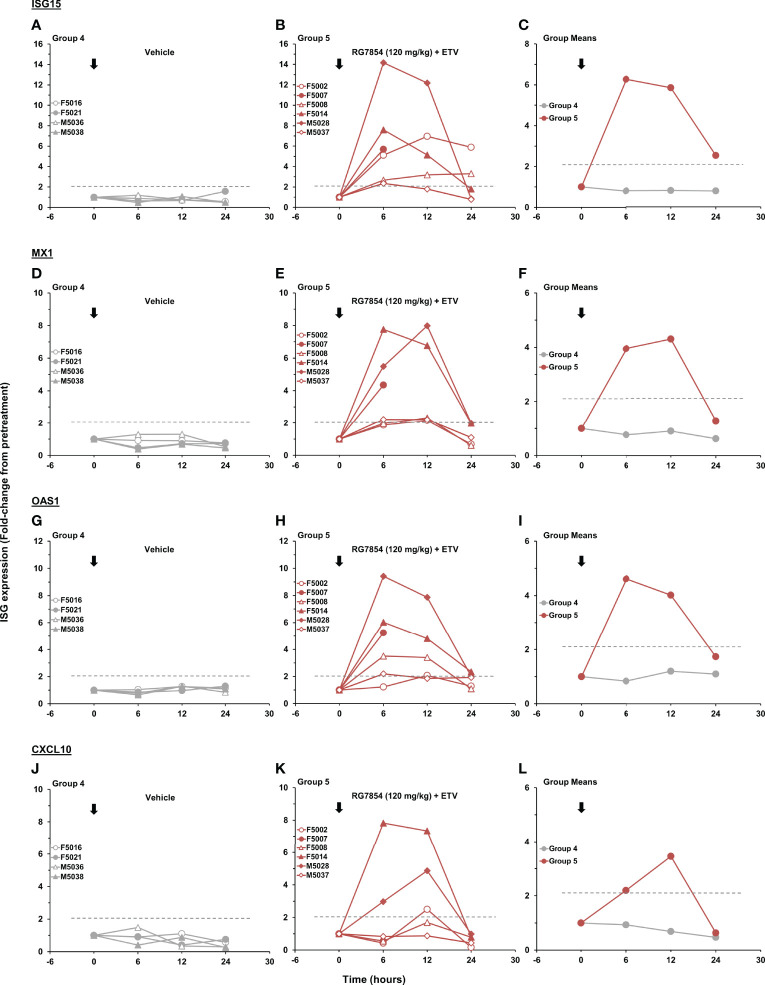
Effect of the first dose of RG7854 together with ETV on peripheral ISG transcription. Kinetics of *ISG15*, *MX1*, *OAS1*, and *CXCL10* gene expression in individual woodchucks prior to (0 hours) and 6-, 12-, and 24-hours post administration of the first dose of **(A, D, G, J)** placebo or **(B, E, H, K)** RG7854 at 120 mg/kg plus ETV in the combination treatment study. **(C, F, I, L)** Group mean ISG transcription levels. The horizontal dotted lines indicate the cutoff for positive gene expression (i.e., ≥2.1-fold increase from the pretreatment baseline). The mean transcription levels of *ISG15*, *MX1*, and *OAS1* in Group 5 were significantly increased compared to Group 4 at 6 and 12 hours, at 6 hours, or at 6 hours, respectively (*P*< 0.05) (Student’s *t*-test). The mean *CXCL10* transcription level in Group 5 was not significantly different to Group 4 (*P*> 0.05).

In line with previous studies on TLR7 agonism (41, 46), RG7854 induced the transcription of ISGs and T-cell attractant chemokines in blood of woodchucks after the first dose in combination with ETV (**Figure 6**). While peak expression of *ISG15*, *MX1*, *OAS1*, and *CXCL10* was observed between 6- and 12-hours post-dose in most animals of Group 5, the transcription magnitude was quite varied. When compared to M5037, the sole Non-Responder, Responders and woodchucks with SVR often had marked expression changes in all four genes. This correlation was only observed partially for F5002 with a SVR, which presented with increased transcription of *ISG15* and *CXCL10* but not of *MX1* and *OAS1*. Since these expression changes were absent in control animals of Group 4 and typically are not observed during ETV monotreatment (46, 67), this indicated on-target activation of TLR7 in woodchucks by RG7854.

### RG7854/ETV Combination Treatment Elicited Virus-Specific T-Cell Responses in the Periphery of Woodchucks With SVR

**Figure 7:**
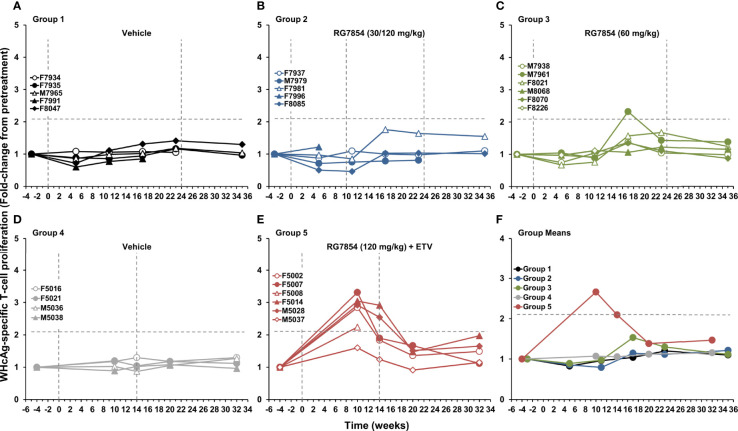
Effect of RG7854, alone and together with ETV, on peripheral WHcAg-specific T-cell response. Kinetics of PBMC proliferation to stimulation with WHcAg-derived peptides of individual woodchucks administered **(A)** placebo or RG7854 at doses of **(B)** 30/120 mg/kg or **(C)** 60 mg/kg in the monotreatment study and **(D)** placebo or **(E)** RG7854 at a dose of 120 mg/kg plus ETV in the combination treatment study. **(F)** Group mean WHcAg-specific T-cell responses. The horizontal dotted lines indicate the cutoff for positive PBMC proliferation (i.e., ≥2.1-fold-change from the pretreatment baseline). The mean WHcAg-specific T-cell response in Groups 2 and 3 was not significantly different to Group 1 (*P*> 0.05) (Student’s *t*-test). The mean WHcAg-specific T-cell response in Group 5 was significantly increased compared to Group 4 at weeks 10 and 14 (*P*< 0.05).

**Figure 8:**
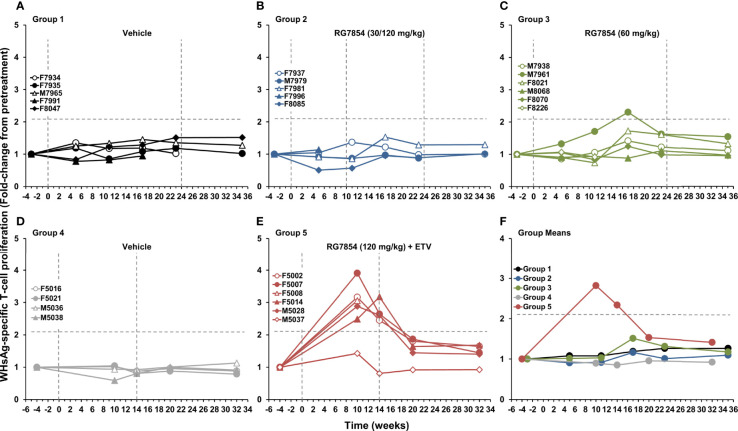
Effect of RG7854, alone and together with ETV, on peripheral WHsAg-specific T-cell response. Kinetics of PBMC proliferation to stimulation with WHsAg-derived peptides of individual woodchucks administered **(A)** placebo or RG7854 at doses of **(B)** 30/120 mg/kg or **(C)** 60 mg/kg in the monotreatment study and **(D)** placebo or **(E)** RG7854 at a dose of 120 mg/kg plus ETV in the combination treatment study. **(F)** Group mean WHsAg-specific T-cell responses. The horizontal dotted lines indicate the cutoff for positive PBMC proliferation (i.e., ≥2.1-fold-change from the pretreatment baseline). The mean WHsAg-specific T-cell response in Groups 2 and 3 was not significantly different to Group 1 (*P*> 0.05) (Student’s *t*-test). The mean WHsAg-specific T-cell response in Group 5 was significantly increased compared to Group 4 at weeks 10 and 14 (*P*< 0.05).

Changes in WHV-specific T-cell responses during RG7854 mono and combination treatment were assessed by stimulating PBMCs of woodchucks with peptides covering the entire WHcAg or WHsAg (**Figures 7**, **8**). RG7854 monotreatment induced transient WHcAg- and WHsAg-specific T-cell responses during treatment only in M7961 of Group 3, a Partial Responder, but not in other Partial Responders, Responders, or woodchucks with SVR in Groups 2 and 3, although an increasing trend to such responses was noted. This pattern was clearly different to RG7854/ETV treatment, as F5002, F5007, F5014, and M5028 of Group 5, all Responders and woodchucks with SVR, except for F5014, presented with pronounced and sometimes long-lasting WHV-specific T-cell responses during treatment that declined after drug withdrawal. Induction and augmentation of WHsAg-specific T-cell responses in these animals apparently correlated with absent surface antigenemia and detectable anti-WHs antibodies in serum (**Figures 3**, **4**). In M5037, the sole Non-Responder, absent WHsAg-specific T-cell response correlated with reduced albeit detectable surface antigen and absent anti-WHs antibodies. Stimulation of PBMCs with LPS as a no-peptide control revealed that the general cell proliferation in woodchucks was not affected by RG7854 mono or combination treatment (**Supplementary Figure 3**). Since ETV monotreatment does not significantly modify cellular responses in woodchucks (67), these results indicated that the inclusion of the NA into the treatment regimen facilitated an enhanced potential of WHV-specific T-cell responses by RG7854.

### RG7854 Treatment, Alone and Together With ETV, Was Safe in Woodchucks

The RG7854 mono and combination treatment regimens were well-tolerated by woodchucks, and there were no signs of overt toxicity based on clinical observations, body weights and temperatures, most hematology and clinical chemistry markers, and necropsy observations. A trend to lower numbers of segmented neutrophils and significantly reduced numbers of platelets were noted in woodchucks of Group 2, especially after the switch to high dose RG7854 treatment, but neutropenia and thrombocytopenia reversed after drug withdrawal (data not shown). Likewise, all animals in Group 5 experienced significant neutropenia and thrombocytopenia during RG7854/ETV combination treatment that reversed immediately after treatment cessation or during treatment, respectively (data not shown).

**Figure 9:**
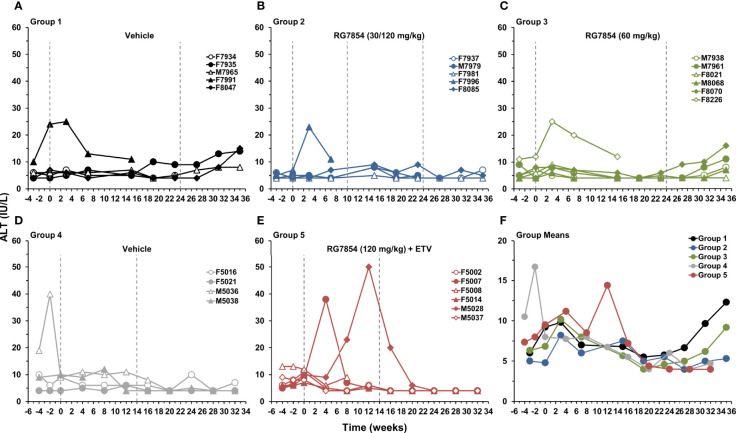
Effect of RG7854, alone and together with ETV, on serum ALT. Kinetics of ALT level in individual woodchucks administered **(A)** placebo or RG7854 at doses of **(B)** 30/120 mg/kg or **(C)** 60 mg/kg in the monotreatment study and **(D)** placebo or **(E)** RG7854 at a dose of 120 mg/kg plus ETV in the combination treatment study. **(F)** Group mean ALT levels. The mean ALT level in Group 2 was significantly reduced compared to Group 1 at week 35 (*P*< 0.05) (Student’s *t*-test). The mean ALT level in Group 5 was not significantly different to Group 4 (*P*> 0.05). IU, international units.

In regard to liver enzymes, F7996 of Group 2 and F8226 of Group 3 had transiently elevated levels of ALT and AST during RG7854 monotreatment, but the rises were comparable to F7991 of Group 1 around the initiation of placebo treatment (**Figure 9** and **Supplementary Figure 4**). The transaminase increases appeared unrelated to RG7854, as they occurred during the progression to end-stage HCC and in parallel to rising GGT levels (**Supplementary Figure 5**), leading to the scheduled euthanasia of these three animals. Other woodchucks in Groups 1 and 3 had gradual increases in ALT, AST, and GGT levels towards the EOS, most likely due to the progression of WHV-induced liver disease. In contrast, F5002, F5007, and M5028 of Group 5 with SVR presented with varied and sometimes marked increases in transaminases during RG7854/ETV combination treatment, in addition to elevations in SDH level (**Supplementary Figure 6**). Elevated liver enzymes coincided with the reductions and subsequent undetectability of serum viremia and antigenemia in these animals, but increases reversed thereafter and normalized during treatment or shortly after drug withdrawal. Furthermore, transient elevations in liver inflammation based on portal and sinusoidal hepatitis scores (**Supplementary Figure 7**) correlated temporally with these liver enzyme increases in woodchucks of Group 5 with SVR, but were also present in F5014, a Responder without SVR, and in M5037, a Non-Responder, albeit to a lesser degree. The rises in ALT, AST, and SDH or liver inflammation, respectively, were comparable to the elevations noted in M5036 and M5038 of Group 4 before the initiation of placebo treatment. Although liver enzyme increases in Group 5 were not significantly different to Group 4, these results suggested that, in contrast to RG7854 monotreatment, the SVR mediated by RG7854/ETV combination treatment was associated with transiently modulated liver enzymes (and likely liver inflammation) in individual woodchucks.

## Discussion

RG7854, an oral double prodrug of the TLR7-specific agonist RO7011785, is developed by F. Hoffmann-La Roche for increasing the HBV cure rate in patients with CHB by immunomodulation *via* TLR7 agonism. The prodrug approach is predicted to widen the therapeutic window of the agonist and to improve its overall tolerability by enhancing bioavailability and limiting intolerability of RO7011785 due to inadvertent TLR7 activation in the gastrointestinal tract (56). For testing therapeutic efficacy, host immune response, and safety, RG7854 was first evaluated in a dose-finding study in woodchucks with chronic WHV infection. Intermediate (60 mg/kg) and high (120 mg/kg) RG7854 dose administration for 24 or 14 weeks, respectively, produced dose-dependent antiviral effects and resulted in a SVR in a total of 3 out of 8 surviving woodchucks. Since these animals seroconverted to anti-WHs antibodies during treatment and WHV DNA and WHsAg remained undetectable at the EOS in week 35, this suggested that a functional cure was induced by RG7854 monotreatment. The SVR achieved in woodchucks is comparable to the durable antiviral effect in an HBV mouse model in which RG7854 dose-dependently reduced the levels of HBV DNA and HBsAg and promoted the emergence of anti-HBs antibodies (57). Because most immunotherapeutic approaches for CHB will be provided to patients on top of standard-of-care with NAs, RG7854 was subsequently evaluated together with ETV in chronic WHV carrier woodchucks. High (120 mg/kg) RG7854 dose administration for 14 weeks in combination with ETV mediated a SVR in 3 out of surviving 5 woodchucks at the EOS in week 32. Based on equal animal numbers with functional cure after RG7854 mono and combination treatment, this indicated that concurrent NA administration did not further increase the therapeutic efficacy of the TLR7 agonist. The SVR in animals undergoing RG7854 combination treatment, including undetectable viral cccDNA in the liver, correlated with the development of innate and adaptive immunity, as determined by the induction of important ISGs at treatment initiation and the proliferation of virus-specific T-cells and production of high-titer, virus-neutralizing antibodies by plasma cells during treatment. Since suppression of WHV replication after initiation of high dose RG7854 mono and combination treatment occurred within 1-6 weeks, and in absence of a ETV monotreatment control group, the contribution of the NA to the therapeutic efficacy could not be differentiated from that of the TLR7 agonist but ETV administration was associated with more robust T- and B-cell responses, as well as transient elevations in liver enzymes and inflammation in some but not all woodchucks.

The SVR produced by RG7854, alone and together with ETV, is clearly different to the antiviral response typically obtained with NAs in woodchucks during comparable treatment durations (48). Although ETV monotreatment was not included as a control in the current study, studies have shown that the ETV-mediated antiviral effect in woodchucks using comparable dosage and treatment durations is transient and that viral relapse occurs after treatment cessation (59, 67), indicating that WHV suppression is dependent on the continued presence of this NA. ETV treatment can modulate WHsAg and WHeAg loads in woodchucks, but similar to patients with CHB undergoing NA treatment (8), it does not mediate loss of these antigens nor induces seroconversion to anti-WHs antibodies (46, 59, 67). The reason for the apparent inability of ETV to enhance the antiviral effect of RG7854 is unknown, but it can be speculated that the immune-mediated WHV suppression by the TLR7 agonist in Responders is rapid and sufficient and cannot be improved by further reduction of mainly viremia levels during parallel NA treatment. This finding is in agreement with the antiviral effect in the above HBV mouse model in which the addition of ETV also did not mediate a greater HBsAg decline than RG7854 alone (68). Since woodchucks are outbred, it can further be speculated that, in addition to genetic factors, Partial Responders and Non-Responders to RG7854 mono and combination treatment developed more severe immunodeficiencies during CHB progression which could not be overcome during the 14- to 24-week treatment duration. Importantly, continuous exposure to viral surface antigen in patients and woodchucks with CHB are implicated in the immunologic tolerance against HBV and WHV at the level of B- and T-cells (3, 14, 29, 30, 48, 69). Future preclinical studies could assess if the therapeutic efficacy of RG7854 is augmentable by administering the TLR7 agonist on top of (prolonged) NA treatment for modelling the most likely treatment scenario in patients. In addition, targeting steps in the HBV lifecycle other than DNA synthesis by the viral polymerase may increase the therapeutic efficacy of RG7854, as indicated in the above HBV mouse model in which parallel treatment with RO7049389, a capsid assembly modulator, produced an antiviral effect that was superior over monotreatment with either compound (68).

Activation of a type-I IFN response in APCs by TLR7 agonists resulting in the production of antiviral cytokines is expected to inhibit HBV replication within hepatocytes by a non-cytolytic mechanism (39). Since assays for the measurement of serum IFN-α were not available, the pharmacodynamic response of woodchucks to the initial RG7854/ETV dose was tested by the IFN-α dependent expression of innate immune genes in blood of these animals. The three ISGs with antiviral effector functions (*ISG15*, *OAS1*, and *MX1*) and the one T-cell-attractant chemokine tested (*CXCL10*) were also recently evaluated in mice and woodchucks during TLR7 agonism (41, 46, 57). Comparable to these studies, ISG and chemokine induction in woodchucks was consistent with TLR7 activation by RG7854 and appeared to correlate partially with the subsequent SVR observed in Responders. Notably, the magnitude of ISG and chemokine induction in woodchucks was similar to those obtained with RG7854 in healthy volunteers at well-tolerated doses of 100 mg or higher, including a high individual variability (56).

Although not determined directly, the transient but sometimes pronounced elevations in liver enzymes and inflammation in Responders to RG7854/ETV combination treatment may indicate that a cytolytic mechanism is further involved in the immune-mediated WHV suppression. Besides antiviral cytokines, the cytotoxic activity of NK- and T-cells during TLR7 agonism were reported to be responsible for the therapeutic efficacy achieved with GS-9620 in woodchucks (41). These predicted mechanisms are supported by enhanced cytokine production of T-cells and increased activation and function of NK-cells in blood of patients with NA-suppressed HBV replication who received add-on GS-9620 treatment (45). Importantly, transient alterations in transaminases during SVR mediated by TLR7 agonism in woodchucks (41, 46) are also present in patients resolving chronic HBV infection while undergoing NA treatment (70). Thus, albeit hepatic flares are typically seen as a clinical perturbation, temporary elevations in liver enzymes (and inflammation) could also be considered as direct evidence for the antiviral activity of TLR7 agonists rather than an unwanted side effect (71).

From the study in the aforementioned HBV mouse model it is hypothesized that RG7854 stimulates TLR7 in pDCs within spleen and lymph nodes but not in the gastrointestinal tract, and that such activated cells prime T- and B-cells for generating an effective immune response against HBV (57). For determining if RG7854-treated woodchucks developed a functional T-cell response, WHV-specific PBMC proliferation was tested longitudinally. In agreement with the recovery of HBV core and polymerase specific T-cells in patients after exposure to low antigen levels and subsequent control of viral replication mediated by NA treatment (72, 73), a WHcAg-specific T-cell response emerged especially in Responders to RG7854/ETV combination treatment. While HBsAg-specific T-cells are usually not retrieved in these patients (73), the recovery of WHsAg-specific T-cells in Responders could indicate that woodchucks with CHB still possess residual, quiescent T-cells directed against this viral antigen similar to young patients with CHB (74), and as expected for self or neoself-reactive T cells. Since WHV-specific T-cells were detected around the time of substantial declines in surface and e antigenemia, this suggested that the removal of tolerizing viral proteins from the system likely retrieved the antiviral functions of helper and cytolytic T-cells and/or activated these cells. The strength and/or duration of these T-cell responses in some Responders correlated with the temporary elevations in transaminases that are considered markers of immune clearance of HBV-infected hepatocytes (70). T-cell responses waned after the loss of WHsAg and WHeAg and were not detected after treatment cessation, indicating that these cells contributed to the RG7854-mediated WHV suppression.

WHV-specific humoral responses were further assessed for determining if RG7854-treated woodchucks developed a functional B-cell response. Responders to monotreatment and especially to RG7854/ETV combination treatment elicited antibodies to WHsAg (and often to WHeAg) that were characterized by remarkably high titers, as also noted for anti-HBs antibodies in the HBV mouse model following treatment with the TLR7 agonist and a capsid assembly modulator (68). Antibody emergence may indicate a reversal of impaired functions of DCs and B-cells in the setting of CHB in patients treated with NAs (30, 75) and in woodchucks treated with TLR agonists, alone and together with ETV (41, 42, 46). Since WHV-specific antibodies were detected immediately after the loss of WHsAg and WHeAg, this suggested again that the elimination of tolerizing viral antigens supported a restoration of B-cell functions and/or activation of these cells, as also reported for patients with NA treatment-induced seroconversion (75). Direct TLR7 stimulation in B-lymphocytes by RG7854 may further lead to polyclonal cell expansion and differentiation into immunoglobulin-secreting plasma cells (76) which could additionally explain the high anti-WHs and anti-HBs titers achieved in woodchucks and mice (68). These virus-neutralizing antibodies are important to prevent reinfection of already infected hepatocytes, as well as *de novo* infection of naïve hepatocytes that emerge during liver replenishment due to natural cell death or cytolytic elimination by T-cells, and of uninfected hepatocytes that arise from non-cytolytic viral elimination by cytokines. Because anti-WHs antibodies persisted until the EOS while WHsAg-specific T-cell responses became undetectable, the durable humoral response is apparently required for the continued suppression of WHV replication by residual virus and/or maintenance of the virus-free status. Altogether, the induction of ISGs and the development of WHV-specific B- and T-cell responses suggested that a crosslink between innate and adaptive immunity was induced by RG7854 mono and combination treatment in Responders with SVR.

RG7854 administration, alone and together with ETV, was not associated with treatment-limiting adverse effects, and repeated dosing for 14 to 24 weeks was well-tolerated by woodchucks. Sustained changes in clinical chemistry and most hematology parameters were not noted; however, RG7854 treatment at the highest dose was associated with thrombocytopenia, and additional neutropenia when combined with ETV. These adverse effects were transient and reversed immediately after treatment cessation or even during treatment, and the incidence and severity was reduced with lower RG7854 doses. The underlying mechanism(s) by which RG7854 causes reductions in neutrophils and platelets in woodchucks are unknown. However, neutropenia and thrombocytopenia appeared woodchuck-specific, since both parameters were not measured for RG7854 treatment in the HBV mouse model (57, 68) and, more importantly, were absent in healthy volunteers administered single and multiple RG7854 doses (56). Moreover, the drug posology for the current clinical phase 2 trial in patients with CHB (i.e., NCT04225715) has a starting RG7854 dose of slightly below 150 mg (56, 77) that is approximately 45-55 times lower than the 100 and 120 mg/kg efficacious doses in mice and woodchucks, and thus is not expected to induce these hematology changes. While the clinical outcome of RG7854 treatment is unknown at this time, it is expected that add-on administration to NA-treated patients in combination with a capsid assembly modulator (RO7049389) or small interfering RNA (RO7445482) will be safe and antiviral efficacious due to the chosen double prodrug approach for improving oral bioavailability and minimizing TLR7 activation in the gastrointestinal tract.

A comparison of therapeutic efficacy achieved in woodchucks with other TLR7 agonists suggested different pharmacokinetics and pharmacodynamics for GS-9620, APR002, and RG7854 (41, 46). As established in the aforementioned HBV mouse model, RG7854 targets spleen and lymph nodes following oral administration and activates pDCs leading to increased numbers of HBV-specific B- and T-cells in these secondary lymphoid organs (57), including upregulated germinal center B-cells (68), but the active metabolite is also detected in liver (data not shown). This overall is different to GS-9620 and APR002 that target gut-associated lymphoid tissues and/or liver after oral administration and intestinal absorption for activating resident pDCs (46, 78). Compared to GS-9620, APR002 is more hepatoselective due to the incorporation of a liver-targeting moiety into the TLR7 pharmacophore (46). Enhanced hepatic and limited systemic exposure of APR002 over GS-9620 is likely due to active uptake and high retainment of the TLR7 agonist in the liver *via* organic-anion-transporting polypeptide (OATP) transporters (46).

In conclusion, RG7854 treatment, alone and together with ETV, produced a SVR and anti-WHs antibody seroconversion in a subset of woodchucks with CHB. By analogy, these results suggest that oral treatment of patients with the double prodrug of the TLR7-specific agonist has the potential to induce sustained immunological control of chronic HBV infection and may present a new therapeutic option in the search for an HBV cure.

## Data Availability Statement

The original contributions presented in the study are included in the article/**Supplementary Material**. Further inquiries can be directed to the corresponding authors.

## Ethics Statement

The animal study was reviewed and approved by Institutional Animal Care and Use Committee of Northeastern Wildlife, Inc. (Harrison, ID) and Georgetown University (Washington, DC).

## Author Contributions

Study concept and design: SW, MV, FR-L, JY, and SM; Acquisition of data: KK, MS, BL, CY, MV, XH, XPH, MM, and BK; Analysis and interpretation of data: SW, MS, GS, LD, MV, FR-L, and SM; Drafting of the manuscript: SW and SM; Critical revision of the manuscript for important intellectual content: SW, KK, MS, GS, LD, BL, CY, MV, FR-L, XH, XPH, MM, BK, JY, and SM; Statistical analysis: GS and SM; Obtained funding: JY; Administrative, technical, or material support: N/A; Study supervision: SW and SM. All authors contributed to the article and approved the submitted version.

## Conflict of Interest

SM has received grants from F. Hoffmann-La Roche, Ltd. and serves occasionally as a paid scientific consultant to Northeastern Wildlife, Inc. GS is employed by Roche, while LD is a former employee of Roche. SW, MV, FR-L, and JY own stock in and are employed by Roche.

The remaining authors declare that the research was conducted in the absence of any commercial or financial relationships that could be construed as a potential conflict of interest.

The authors declare that this work was funded by F. Hoffmann-La Roche, Ltd. (https://www.roche.com) who played a role in the design of the study, collection of data, analysis and interpretation of data, preparation of the manuscript, and in the decision to submit the manuscript for publication.

## Publisher’s Note

All claims expressed in this article are solely those of the authors and do not necessarily represent those of their affiliated organizations, or those of the publisher, the editors and the reviewers. Any product that may be evaluated in this article, or claim that may be made by its manufacturer, is not guaranteed or endorsed by the publisher.

## Abbreviations

ALT, alanine aminotransferase; Anti-HBs, antibodies to hepatitis B virus surface antigen; Anti-WHe, antibodies to woodchuck hepatitis virus e antigen; Anti-WHs, antibodies to woodchuck hepatitis virus surface antigen; APC, antigen-presenting cell; APR002, a TLR7 agonist developed by Apros Therapeutics; AST, aspartate aminotransferase; cccDNA, covalently-closed circular DNA; CHB, chronic hepatitis B; CXCL10, IFN-γ induced protein 10 (IP-10); DC, dendritic cell; EOS, end of the study; ETV, entecavir; F, female; ge, genome equivalents or copy numbers; GGT, gamma-glutamyl transferase; GS-9620, a TLR7 agonist developed by Gilead Sciences; HBsAg, hepatitis B virus surface antigen; IFN, interferon; ISG, interferon-stimulated gene; ISG15, IFN-induced 17 kDa protein; LPS, lipopolysaccharide; M, male; MX1, IFN-induced guanosine triphosphate-binding protein; NA, nucleos(t)ide analogue; ND, not determined; NK, natural killer; NR, Non-Responders; OAS1, 2’-5’-oligoadenylate synthetase 1; PBMC, peripheral blood mononuclear cell; PR, Partial Responders; PRR, pathogen recognition receptor; QOD, every other day; R, Responders; RG7854, an oral double prodrug of the TLR7-specific agonist RO7011785; RI, replicative intermediate; RO7011785, a TLR7 agonist developed by F. Hoffmann-La Roche, Ltd.; RO7049389, capsid assembly modulator developed by F. Hoffmann-La Roche, Ltd.; SDH, sorbitol dehydrogenase; StdU, standard units; SVR, sustained antiviral response; TLR, toll-like receptor; WHcAg, woodchuck hepatitis virus core antigen; WHeAg, woodchuck hepatitis virus e antigen; WHsAg, woodchuck hepatitis virus surface antigen; WHV, woodchuck hepatitis virus.
