# Supplementary material for: Toll-Like Receptor 7 Agonist RG7854 Mediates Therapeutic Efficacy and Seroconversion in Woodchucks With Chronic Hepatitis B
Source: Front Immunol. 2022 May 23;13:884113. doi: 10.3389/fimmu.2022.884113 (PMC9169629; doi:10.3389/fimmu.2022.884113)
Supplement: Supplementary file 1 [file DataSheet_1.docx]

Supplementary Material

## 1 Supplementary Tables

**Supplementary Table 1. Allocation of woodchucks to treatment groups.**

| **Study** | **Group** | **Treatment** | **N** | **Animal Identification** |
| --- | --- | --- | --- | --- |
| Monotreatment | 1 | Vehicle (po, QOD) | 5 | F7934, F7935, M7965, F7991, F8047 |
|  | 2 | RG7854 (30/120 mg/kg, po, QOD)**^a^** | 5 | F7937, M7979, F7981, F7996, F8085 |
|  | 3 | RG7854 (60 mg/kg, po, QOD) | 6 | M7938, M7961, F8021, M8068, F8070, F8226 |
| Combination treatment | 4 | Vehicle (po, QOD and QD) | 4 | F5016, F5021, M5036, M5038 |
|  | 5 | RG7854 (120 mg/kg, po, QOD) + ETV (0.1 mg/kg, po, QD) | 6 | F5002, F5007, F5008, F5014, M5028, M5037 |

**^a^**The dose level in Group 2 was increased to 120 mg/kg during week 10. Abbreviations: N, number; po, oral; QOD, every other day; QD, every day; F, female; M, male.

**Supplementary Table 2. Woodchuck-specific primers and probes used for the analysis of peripheral ISG transcription during RG7854/ETV combination treatment.**

| **Gene** | **Primers and Probe** | **Sequence** |
| --- | --- | --- |
| ISG15 | F | 5'- CTGTTCTGGCTGAGCTTCG -3' |
|  | R | 5'- GCAGGTTCAGAAACACAGTGC -3' |
|  | P | 5'- GGGAGTATGGACTCACCCCT -3' |
| MX1 | F | 5'- GCTGGACGAAGGAAAGGC -3' |
|  | R | 5'- AGTGGCAGGGATTTACAGATG -3' |
|  | P | 5'- AGCTCAGTGGTCAGTCTCTCCGC -3' |
| OAS1 | F | 5'- AGTTCACGATGGTCCAATCC -3' |
|  | R | 5'- GTGCCAGGGCATCAAAAG -3' |
|  | P | 5'- GCTTCGTGCTGAGTTCCTCT -3' |
| CXCL10 | F | 5'- AAAAAGAGCGGGGAGAAGAG -3' |
|  | R | 5'- GGAGCCCTTTTAGACCTTTCAT -3' |
|  | P | 5'- TCCAGAATCTAAAGCCATCAAGA -3' |
| 18S rRNA | F | 5’- GTAACCCGTTGAACCCCATT -3’ |
|  | R | 5’- GGGACTTAATCAACGCAAGC -3’ |
|  | P | 5’- GCAATTATTCCCCATGAACG -3’ |

Abbreviations: F, forward primer; R, reverse primer; P, probe.

## 2 Supplementary Figures


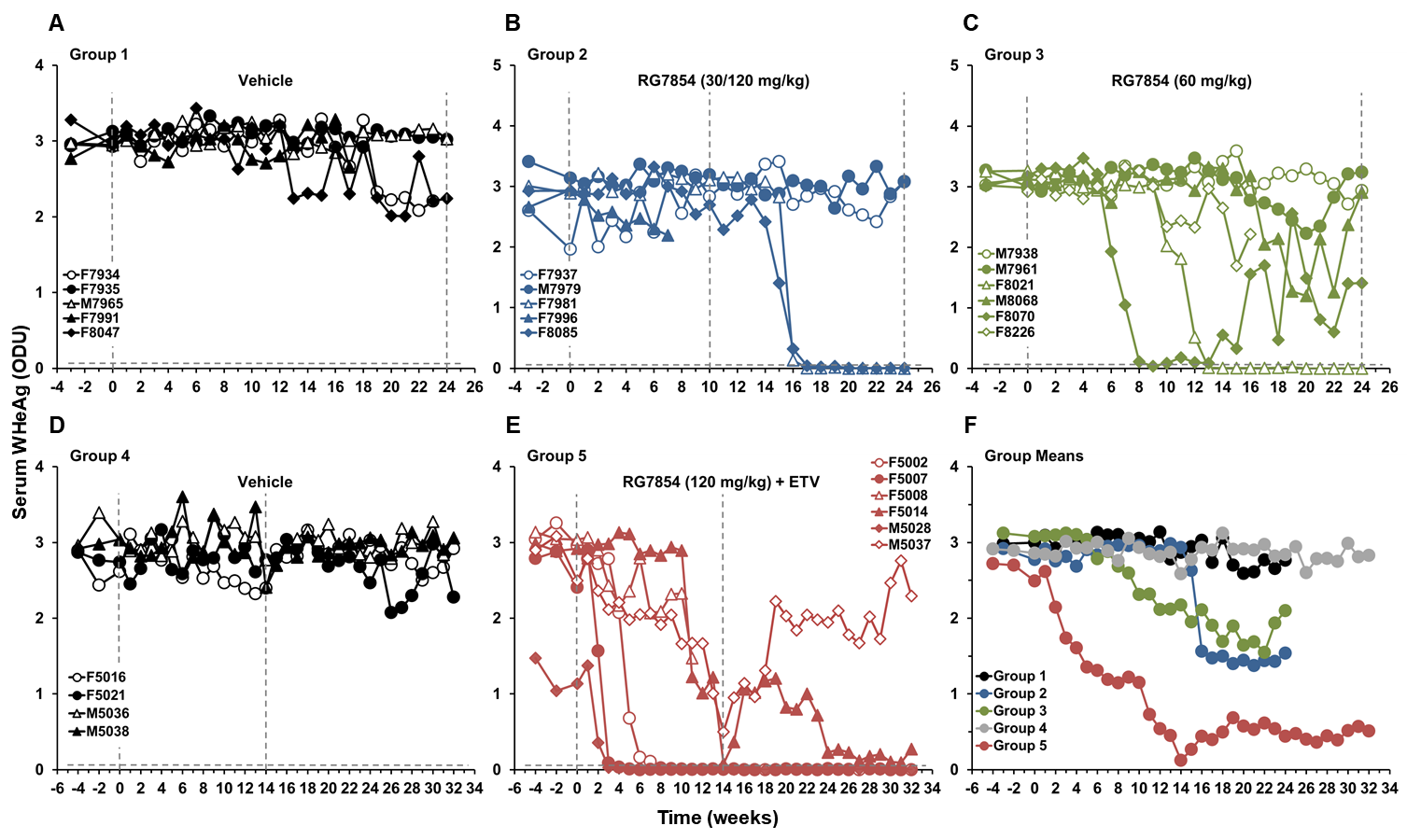


**Supplementary Figure 1. Effect of RG7854, alone and together with ETV, on serum e antigenemia levels.** Kinetics of WHeAg load in individual woodchucks administered **(A)** placebo or RG7854 at doses of **(B)** 30/120 mg/kg or **(C)** 60 mg/kg in the monotreatment study and **(D)** placebo or **(E)** RG7854 at a dose of 120 mg/kg plus ETV in the combination treatment study. **(F)** Group mean WHeAg loads. The outer vertical dotted lines represent the duration of 24-week monotreatment or 14-week combination treatment, while the inner vertical dotted line represents the switch from 30 to 120 mg/kg RG7854 in Group 2 during week 10 in this and the following figures. The horizontal dotted lines indicate the detection limit for WHeAg (i.e., ≤0.060 ODU). The mean WHeAg loads in Group 2 and Group 3 were not significantly different to Group 1 (*P*> 0.05) (Student’s *t*-test). Compared to Group 4, the mean WHeAg load in Group 5 was significantly reduced during weeks 12-18, at weeks 20 and 21, and during weeks 23-32 (*P*< 0.05). Abbreviation: ODU, optical density units.


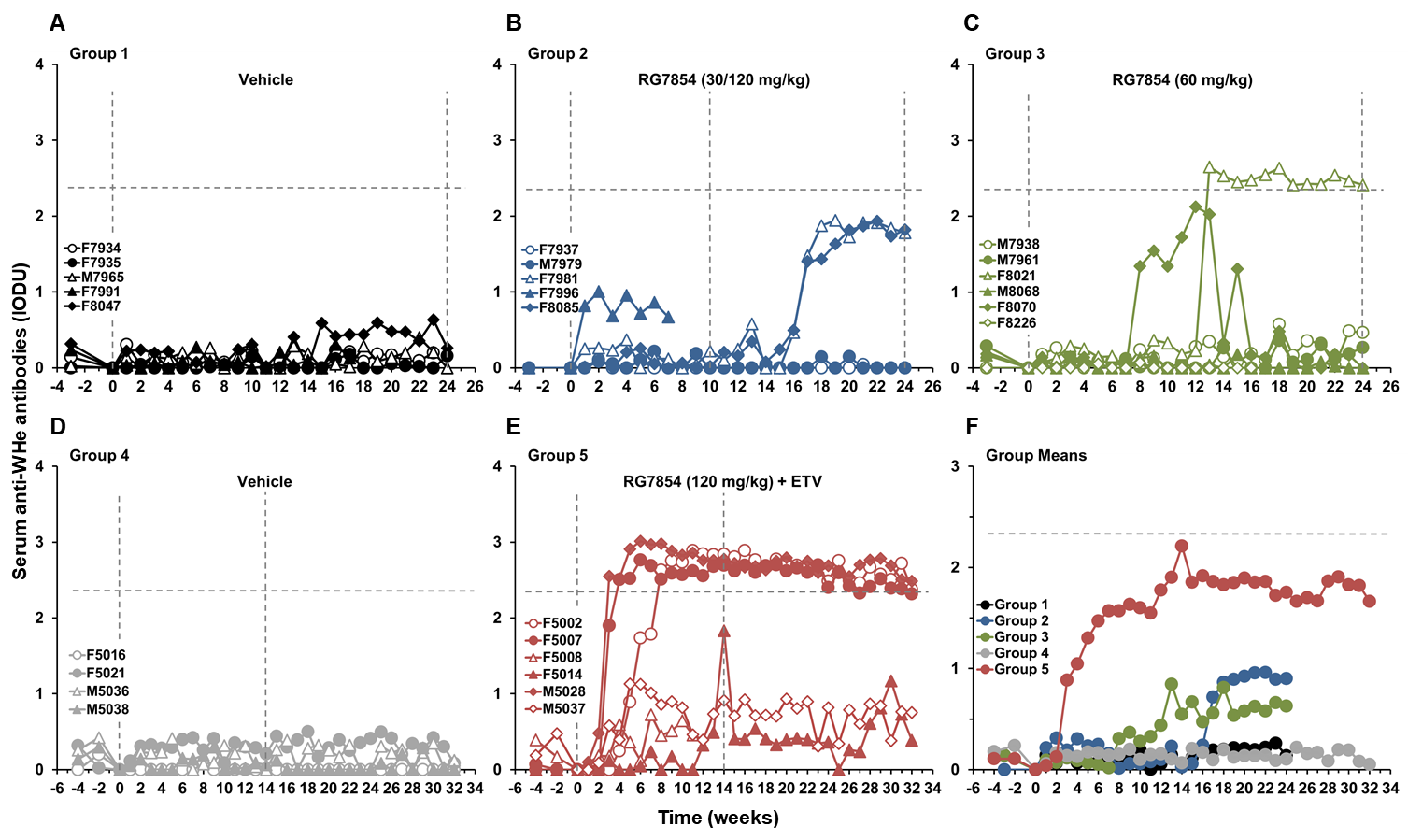


**Supplementary Figure 2. Effect of RG7854, alone and together with ETV, on the elicitation of serum antibodies to WHeAg.** Kinetics of anti-WHe antibody level in individual woodchucks administered **(A)** placebo or RG7854 at doses of **(B)** 30/120 mg/kg or **(C)** 60 mg/kg in the monotreatment study and **(D)** placebo or **(E)** RG7854 at a dose of 120 mg/kg plus ETV in the combination treatment study. **(F)** Group mean anti-WHe antibody levels. The horizontal dotted lines indicate the detection limit for anti-WHe antibodies (i.e., ≥2.33 ODU). The mean anti-WHe antibody levels in Group 2, Group 3, and Group 5 were not significantly different to Group 1 or Group 4, respectively (*P*> 0.05) (Student’s *t*-test). Abbreviation: ODU, optical density units.


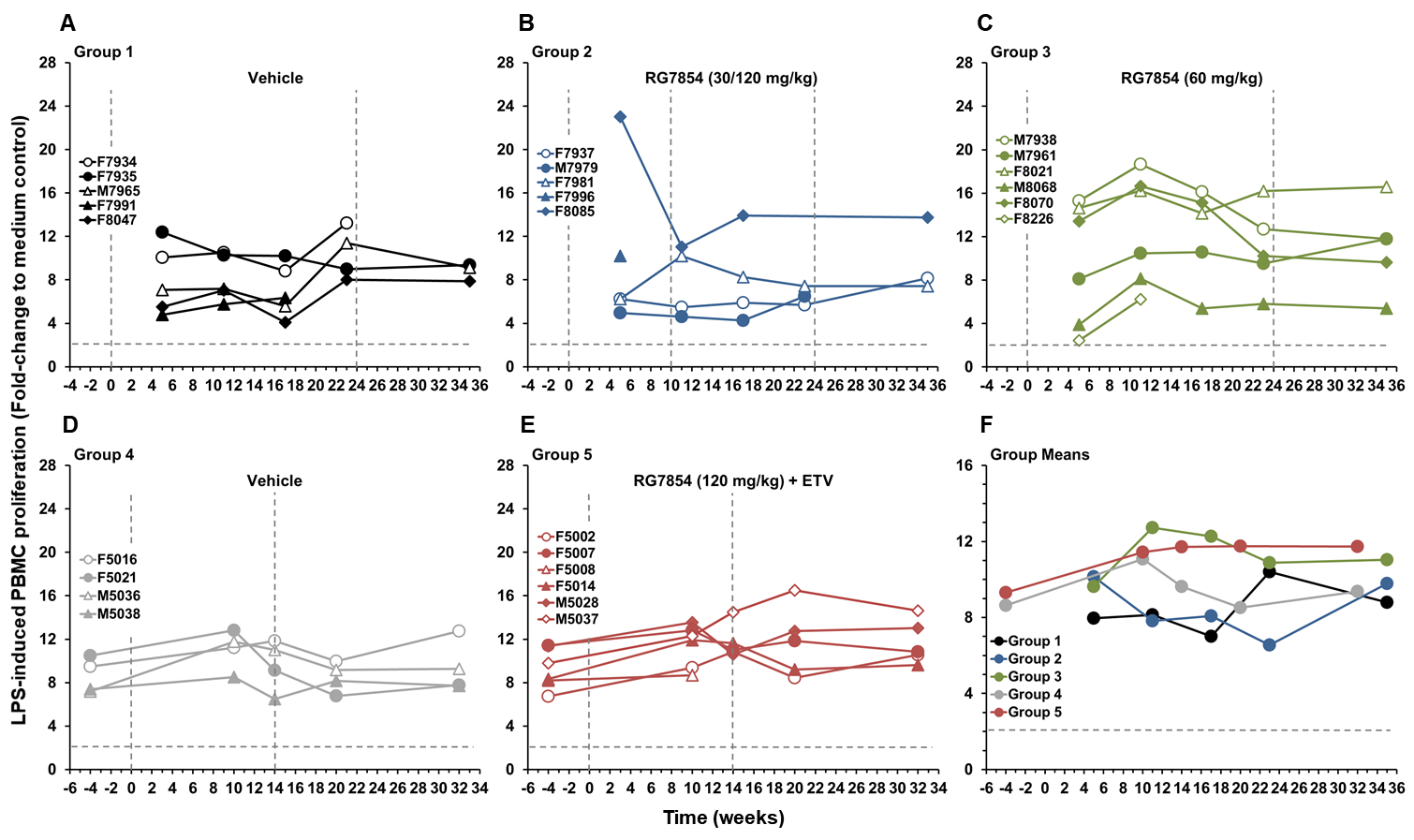


**Supplementary Figure 3. No effect of RG7854, alone and together with ETV, on LPS-induced PBMC proliferation.** Kinetics of PBMC proliferation to stimulation with the no-peptide control LPS of individual woodchucks administered **(A)** placebo or RG7854 at doses of **(B)** 30/120 mg/kg or **(C)** 60 mg/kg in the monotreatment study and **(D)** placebo or **(E)** RG7854 at a dose of 120 mg/kg plus ETV in the combination treatment study. **(F)** Group mean PBMC proliferation induced by LPS. The horizontal dotted lines indicate the cutoff for positive PBMC proliferation (i.e., ≥2.1-fold-change from the medium control stimulation at each time point). The mean PBMC proliferation induced by LPS in Group 2 and Group 3 was significantly reduced or increased at weeks 23 or 17, respectively, when compared to Group 1 (*P*< 0.05) (Student’s *t*-test). Compared to Group 4, the mean PBMC proliferation induced by LPS in Group 5 was not significantly different (*P*> 0.05).


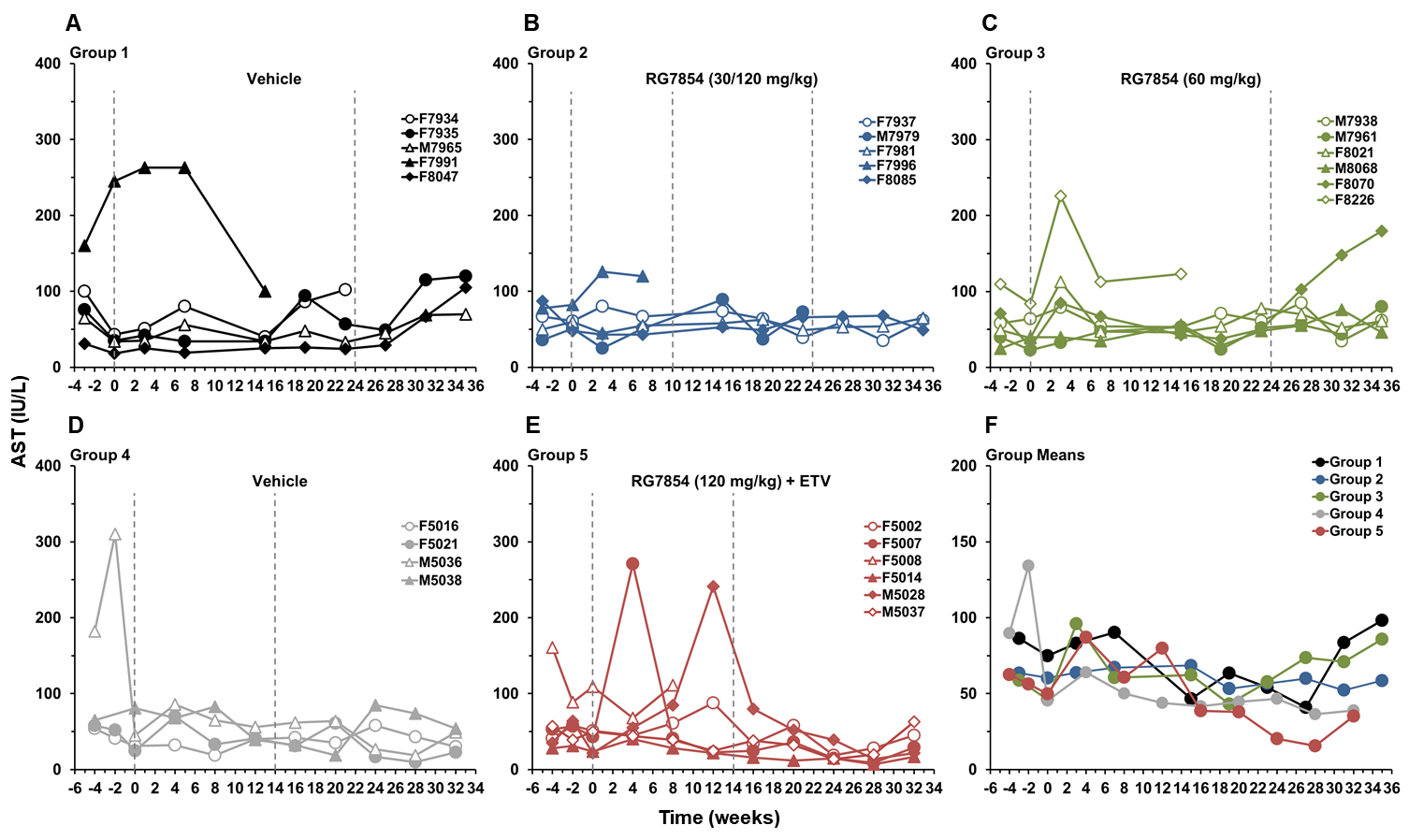


**Supplementary Figure 4. Effect of RG7854, alone and together with ETV, on serum AST.** Kinetics of AST level in individual woodchucks administered **(A)** placebo or RG7854 at doses of **(B)** 30/120 mg/kg or **(C)** 60 mg/kg in the monotreatment study and **(D)** placebo or **(E)** RG7854 at a dose of 120 mg/kg plus ETV in the combination treatment study. **(F)** Group mean AST levels. The mean AST level in Group 3 was significantly increased compared to Group 1 at week 27 (*P*< 0.05) (Student’s *t*-test). Compared to Group 4, the mean AST level in Group 5 was not significantly different (*P*> 0.05). Abbreviation: IU, international units.


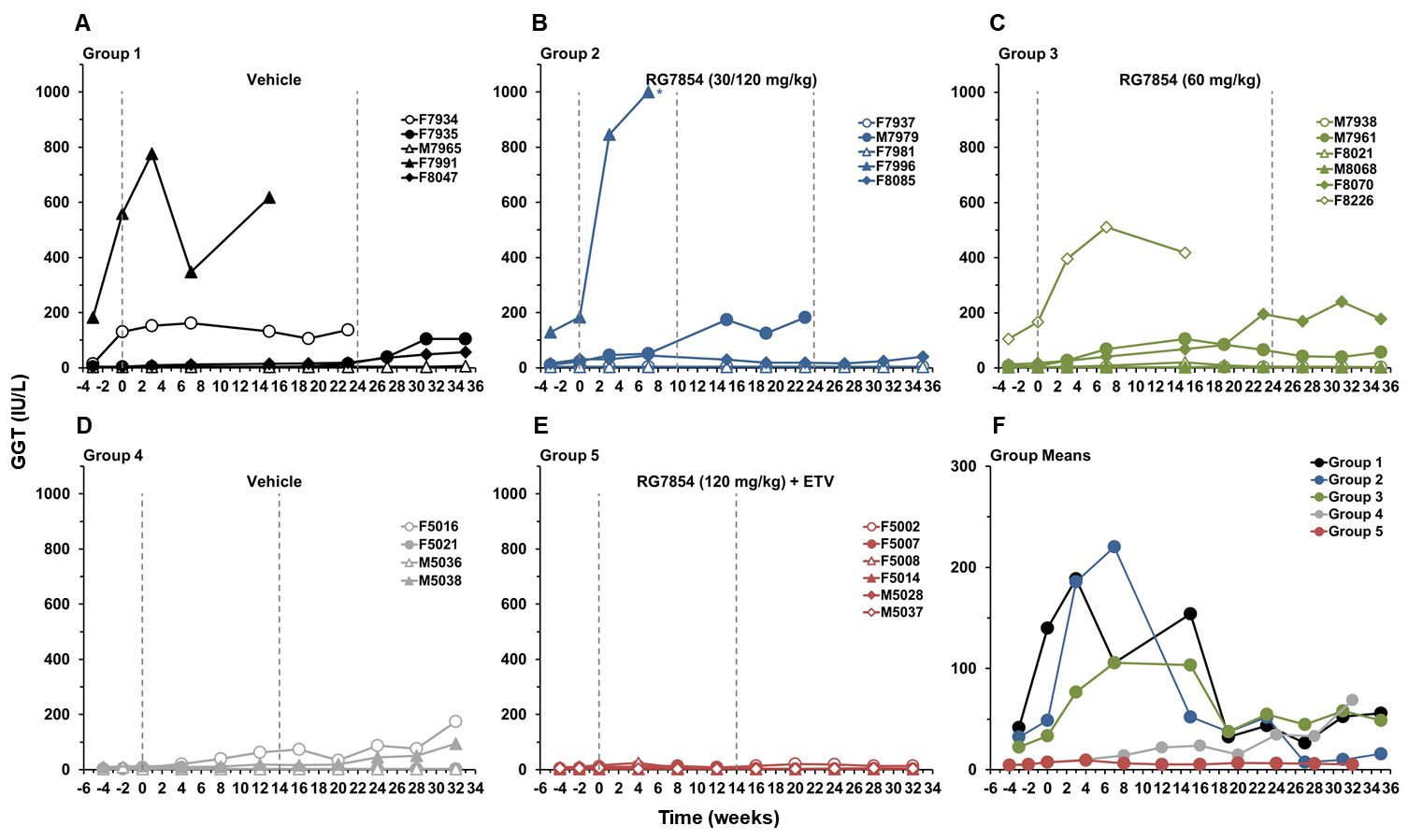


**Supplementary Figure 5.** **Effect of RG7854, alone and together with ETV, on serum GGT.** Kinetics of GGT level in individual woodchucks administered **(A)** placebo or RG7854 at doses of **(B)** 30/120 mg/kg or **(C)** 60 mg/kg in the monotreatment study and **(D)** placebo or **(E)** RG7854 at a dose of 120 mg/kg plus ETV in the combination treatment study. **(F)** Group mean GGT levels. The mean GGT levels in Group 2, Group 3, and Group 5 were not significantly different to Group 1 or Group 4, respectively (*P*> 0.05) (Student’s *t*-test). *, Animal F7996 had a GGT level of 1,148 IU at week 7. Abbreviation: IU, international units.


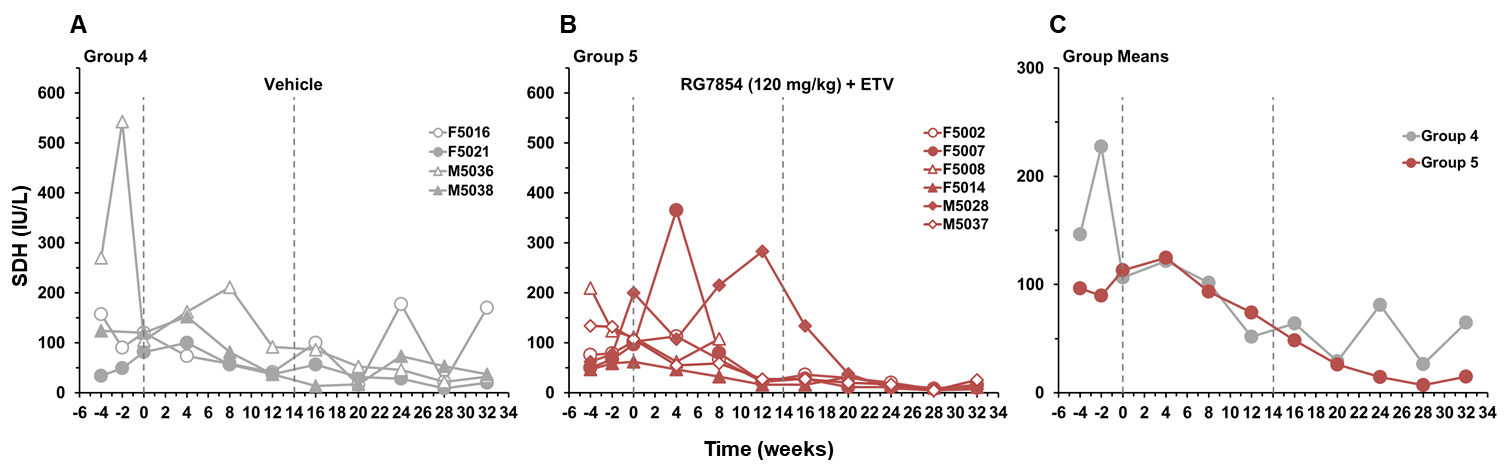


**Supplementary Figure 6. Effect of RG7854 together with ETV on serum SDH.** Kinetics of SDH level in individual woodchucks administered **(A)** placebo or **(B)** RG7854 at a dose of 120 mg/kg plus ETV in the combination treatment study. **(C)** Group mean SDH levels. The mean SDH level in Group 5 was not significantly different to Group 4 (*P*> 0.05) (Student’s *t*-test). Abbreviation: IU, international units.


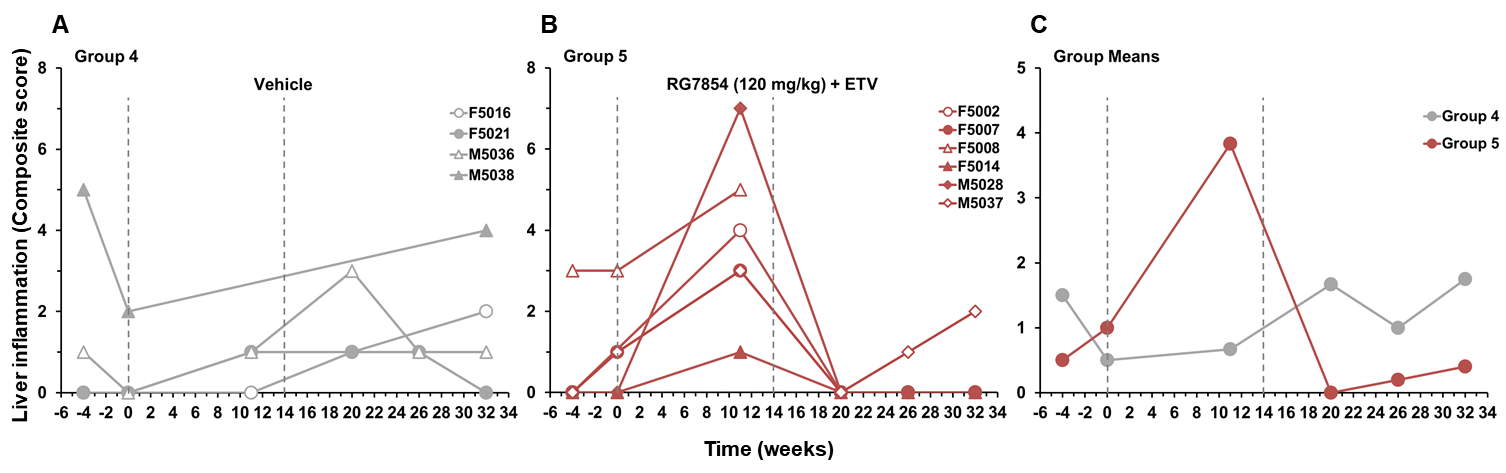


**Supplementary Figure 7. Effect of RG7854 together with ETV on liver inflammation.** Kinetics of portal and sinusoidal hepatitis composite score in individual woodchucks administered **(A)** placebo or **(B)** RG7854 at a dose of 120 mg/kg plus ETV in the combination treatment study. **(C)** Group mean composite scores. The composite score was derived from the mean of the portal hepatitis score (1-4 portal tracts examined) combined with the lobular sinusoidal hepatitis score. The mean composite score in Group 5 was significantly increased and reduced compared to Group 4 at week 11 or week 20, respectively (*P*< 0.05) (Student’s *t*-test). A composite score of 0 indicates absent, >0-2 indicates mild, >2-4 indicates moderate, >4-6 indicates marked, and >6 indicates severe liver inflammation.
